# Characterization of Sex Determination and Sex Differentiation Genes in *Latimeria*

**DOI:** 10.1371/journal.pone.0056006

**Published:** 2013-04-25

**Authors:** Mariko Forconi, Adriana Canapa, Marco Barucca, Maria A. Biscotti, Teresa Capriglione, Francesco Buonocore, Anna M. Fausto, Daisy M. Makapedua, Alberto Pallavicini, Marco Gerdol, Gianluca De Moro, Giuseppe Scapigliati, Ettore Olmo, Manfred Schartl

**Affiliations:** 1 Dipartimento di Scienze della Vita e dell'Ambiente, Università Politecnica delle Marche, Ancona, Italy; 2 Dipartimento di Biologia Strutturale e Funzionale, Università Federico II, Napoli, Italy; 3 Dipartimento per l'Innovazione nei Sistemi Biologici, Agroalimentari e Forestali, Università della Tuscia, Viterbo, Italy; 4 Faculty of Fisheries and Marine Science, University of Sam Ratulangi, Manado, Indonesia; 5 Dipartimento di Scienze della Vita, Università di Trieste, Trieste, Italy; 6 Physiological Chemistry, Biocenter, University of Wuerzburg, Wuerzburg, Germany; University of Illinois at Chicago, United States of America

## Abstract

Genes involved in sex determination and differentiation have been identified in mice, humans, chickens, reptiles, amphibians and teleost fishes. However, little is known of their functional conservation, and it is unclear whether there is a common set of genes shared by all vertebrates. Coelacanths, basal Sarcopterygians and unique “living fossils”, could help establish an inventory of the ancestral genes involved in these important developmental processes and provide insights into their components. In this study 33 genes from the genome of *Latimeria chalumnae* and from the liver and testis transcriptomes of *Latimeria menadoensis*, implicated in sex determination and differentiation, were identified and characterized and their expression levels measured.

Interesting findings were obtained for *GSDF*, previously identified only in teleosts and now characterized for the first time in the sarcopterygian lineage; *FGF9*, which is not found in teleosts; and *DMRT1*, whose expression in adult gonads has recently been related to maintenance of sexual identity. The gene repertoire and testis-specific gene expression documented in coelacanths demonstrate a greater similarity to modern fishes and point to unexpected changes in the gene regulatory network governing sexual development.

## Introduction

Two major processes take place in sexual development: sex determination and sex differentiation. The former process determines whether the bipotential primordium will develop into a testis or an ovary; the latter takes place after sex determination and involves the actual development of testes or ovaries from the undifferentiated gonad [1]. Sex determination is considered as a default pathway or as suppression thereof and initiation of the opposite pathway; in contrast, sex differentiation seems to result from the antagonistic relationship among the genes influencing testis or ovary development [2], [3]. Recently it has emerged that sex-specific mechanisms, which are critical to maintaining the male or female identity of the testis and ovary, also operate in adult mammalian gonads [4]–[6]. Other organs besides the gonads may also acquire elaborate male- and female-specific differences. In vertebrates—with the possible exception of birds [7]—such secondary sexual traits are generally believed to be instructed exclusively by the developing testis or ovary through sex steroids, whereas in invertebrates each somatic cell seems to have an inherent sexual identity [8]. Compared with eutherian mammals, sex steroids and the proteins involved in their metabolism and binding play an earlier role in the sex differentiation process of fish, amphibians, reptiles, birds, and marsupials [9]–[20].

In vertebrates sexual development is determined by two main factors: either the genetic makeup of the individual or the environment, through the influence of temperature during development, nutrients, pH, etc [21]–[23]. It has been demonstrated that in mammals the consecutive processes of sex determination, gonad differentiation and identity maintenance are brought about by a complex network of transcription factor interactions and signalling molecules; a master regulator upstream then directs the network towards male or female [24]. The male-determining gene in most mammals is the Y chromosome *SRY* gene, which however has only been detected in placental mammals [25]. In chickens (and possibly all birds) the master regulator of sexual development is *Dmrt1*; its homologues are *dmrt1bY* (or *DMY*) in the Japanese ricefish (medaka, *Oryzias latipes*) [26], [27]; and *DM-W* in the frog *Xenopus laevis* [28]. In several fish species this function is served by *gonadal soma-derived factor* (*GSDF*) [29], *anti-Müllerian hormone* (*AMH*) [30], *anti-Müllerian hormone receptor* (*AMHR2*) [31], or other genes.

In contrast to the variety of upstream sex determinants, genome-wide studies and homology cloning in teleost fishes, amphibians, reptiles and birds have suggested that the downstream components of the network have a conserved function. This has inspired the paradigm that in sex determination during evolution “masters change, slaves remain” [32]–[34]. However, it is unclear how far back in the evolutionary history this applies and in particular when and how the vertebrate sex regulation network evolved and whether the relevant genes represent an ancient, conserved mechanism or else they were repeatedly and independently recruited to the process.

The unique opportunity to examine high-quality RNA from the Indonesian coelacanth *Latimeria menadoensis* for transcriptome analysis of testis and liver tissue, and the availability of the whole genome sequence of the African coelacanth *Latimeria chalumnae*, enabled us to gain insights into a “living fossil” that is held to be among the nearest living relatives of tetrapods.

**Figure 1:**
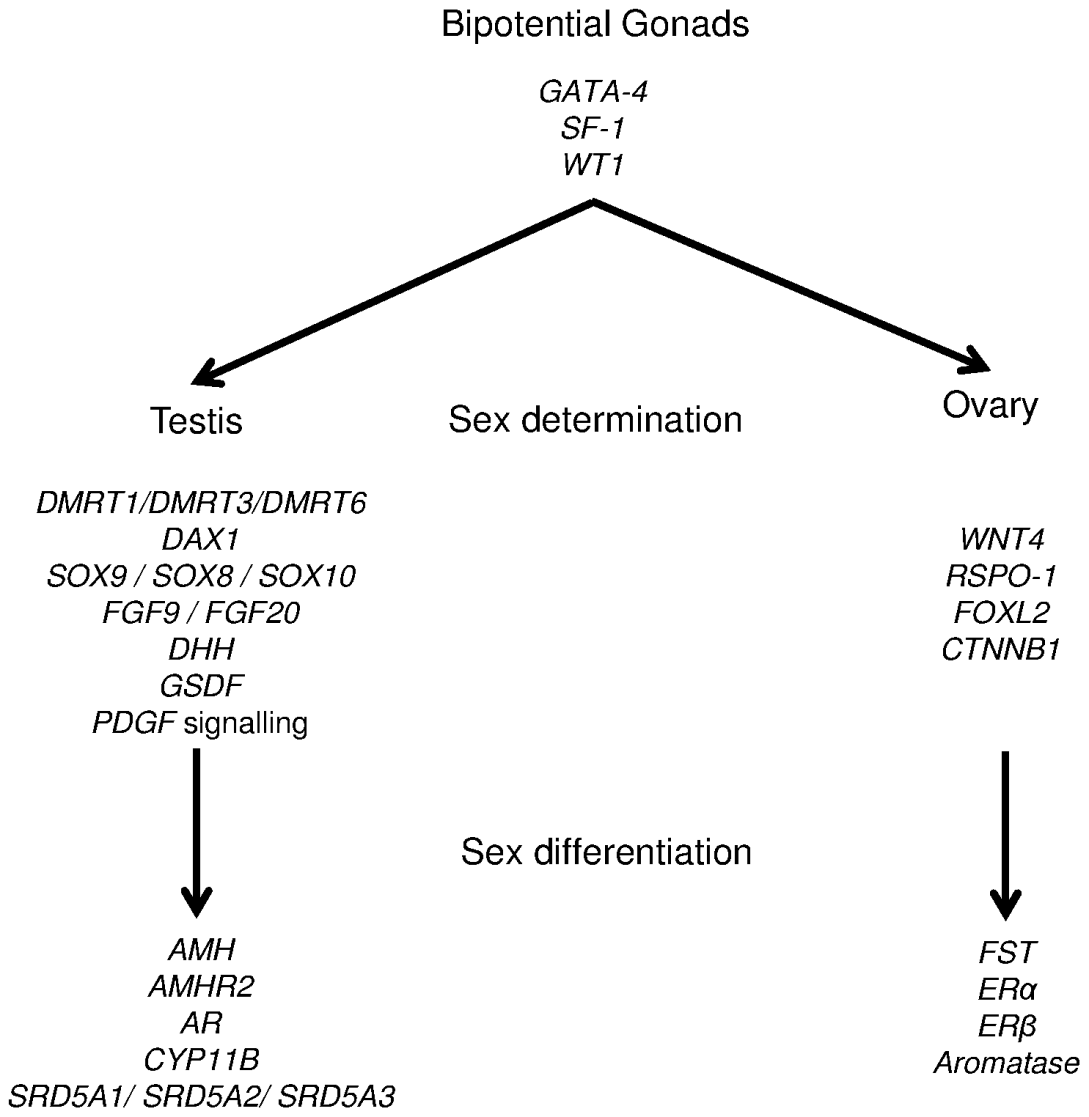
Genes involved in sexual development.

The genes involved in the regulatory network of sexual development described so far come mainly from mammalian studies and can be divided into functional groups as follows: 1) genes required for bipotential gonad development [*Wilm's tumour suppressor-1* (*WT1*), *steroidogenic factor-1* (*SF-1*), and *GATA-binding protein 4* (*GATA-4*)]; 2) genes involved in male sex determination [*double sex and mab-3 related transcription factor 1* (*DMRT1*), *SRY-related box 9* (*SOX9*), *dosage-sensitive sex-reversal-adrenal hypoplasia congenital-critical region of X chromosome, gene 1* (*DAX1*), *fibroblast growth factor 9* (*FGF9*), and *desert hedgehog* (*DHH*)]; 3) genes involved in male sex differentiation [*AMH, AMHR2*, and *androgen receptor* (*AR*)]; 4) genes involved in female sex determination [*Wingless-type MMTV integration site family member 4* (*WNT4*), *R-spondin-1* (*RSPO-1*), *catenin β-1* (*CTNNB1*), *forkhead box transcription factor L2* (*FOXL2*), and *follistatin* (*FST*)]; 5) genes involved in female sex differentiation [*aromatase* (also known as *Cyp19A1* or *P450arom*), *oestrogen receptor α* (*ERα*), and *oestrogen receptor β* (*ERβ*)] (Figure 1).

These 22 genes and 11 other genes [*DMRT3*, *DMRT6*, *GSDF*, *platelet-derived growth factors* (*PDGF*) *α* and *β* and their receptors (*PDGFRα*, *PDGFRβ*), *11β-hydroxylase* (*CYP11B*), and *5α-reductase 1*, *2*, and *3* (*SRD5A1*, *SRD5A2*, *SRD5A3*)], whose involvement in sex development has been documented [35]–[47], were sought in the *L. chalumnae* genome and in the transcriptome of *L. menadoensis*; subsequently their expression levels were measured in the liver and testis of an adult specimen of *L. menadoensis*.

The coelacanth gene repertoire and expression profiles were much more similar to those of modern fish than to those of tetrapods, although they may also represent an intermediate condition; these data unexpectedly suggest that the major evolutionary changes accompanying the transition to terrestrial life were also involved in gonad development.

## Methods

The genome of the African coelacanth *L. chalumnae* has recently been sequenced (project accession PRJNA56111) [48] and is available in the framework of the whole genome shotgun (WGS) sequencing project at http://www.ncbi.nlm.nih.gov and http://www.ensembl.org. The transcriptome of its Indonesian congener, *L. menadoensis*, has been described by Pallavicini and colleagues [49] and Canapa and co-workers [50]. Briefly, an adult male specimen of *L. menadoensis* weighing 27 kg was caught in a shark net near Talise island, Indonesia [51]. Liver and testis were collected immediately after death and preserved in RNAlater (Applied Biosystems, Warrington, UK). A good quality RNA samples, extracted using Trizol Reagent (Ambion/Life Technologies, Carlsbad, CA) following the manufacturer's instructions (RNA integrity number was 7.0 for testis and 6.6 for liver), were used to generate cDNA libraries for transcriptome sequencing on the Illumina Genome Analyzer II platform (Illumina, San Diego, CA, USA). After filtering high-quality reads, removing reads containing primer/adaptor sequences, and trimming read length, the Illumina 100-bp paired-end reads were assembled on a 4-core server (72GB RAM). CLC Genomic Workbench 4.5.1 (CLC Bio, Katrinebjerg, Denmark) and Trinity [52] were used for *de novo* assembly of short reads. Contigs confirmed and improved by both methods were pooled in a high-quality set.

To identify the coelacanth homologues of the genes involved in sexual development, the corresponding *Xenopus tropicalis, Gallus gallus, Danio rerio* and *Homo sapiens* sequences were BLASTed on the *L. menadoensis* transcript dataset. The identity of each retrieved putative transcript was confirmed through NCBI BLAST by homology. BLASTx analyses allowed transcript completeness to be established (coding sequences, CDSs).

The *L. menadoensis* sequences were then BLASTed against the WGS dataset of *L. chalumnae*, to identify the genomic scaffolds of the African coelacanth containing them. Species divergence was calculated with PAUP on the matching sequences as p-distance percentage; the Ka/Ks ratio was calculated with KaKs_calculator [53] using the Nei and Gojobori method [54]. The synonymous distance was calculated using MEGA5 [55] by applying the uncorrected modified Nei and Gojobori method [56] to the concatenated CDSs, aligned with ClustalW2 (http://www.ebi.ac.uk/Tools/msa/clustalw2/; [57]).

The predicted transcripts of *L. chalumnae* were collected from ENSEMBL (http://www.ensembl.org/Latimeria_chalumnae/Info/Index). The *GSDF* CDS was obtained manually by aligning *L. menadoensis* transcripts to the *L. chalumnae* genome; *FGF9*, not found in the transcriptome and not annotated in ENSEMBL, was obtained manually by BLASTing annotated amino acid sequences of other species to the *L. chalumnae* WGS. *GSDF* and *FGF9* putative transcripts were confirmed by homology through NCBI BLAST.

*L. chalumnae* and *L*. *menadoensis* transcripts were compared by ClustalW2 alignment; a graphical representation of each sequence pair is reported in Figures S1A and S1B.

Gene ontology (GO) terms involved in sex determination and sex differentiation (GO0007530 and GO0007548, respectively) were selected and *L. menadoensis* orthologues to *D. rerio*, *X. tropicalis*, *G. gallus*, *Canis familiaris*, *Bos taurus*, *Sus scrofa*, *Mus musculus*, *Rattus norvegicus* and *H. sapiens* counterparts counted.

*L. menadoensis* liver and testis gene expression levels were calculated using the CLC Genomic Workbench 4.5.1 by mapping paired reads from the transcriptome on the assembled transcripts, and given as Fragments Per Kilobase of exon per Million sequenced fragments (FPKM). The lack of some transcripts in the assembled transcriptome may depend on poor gene expression, hence on the limited number of reads, which prevented assembly of a contig. In such cases ENSEMBL gene predictions were used to determine absence or low expression taking into account the predicted transcripts. The FPKM value is therefore still a function of transcript length rather than gene length. The FPKM value was calculated for *DMRT3*, *FOXL2*, *aromatase*, *WNT4*, and *CYP11B* on ENSEMBL transcript predictions as well as on the inferred sequence of *L. chalumnae FGF9*.

Besides genes expected to be involved in sexual development, the expression levels of some house-keeping genes, i.e. *phosphoglycerate kinase* (*PGK*), *heat shock protein class B* (*HSPCB*), and the ribosomal proteins *RPS27*, *RPL19*, *RPL11*, *RPL32*, chosen according to Eisenberg and Levanon [58], were also evaluated.

Correct assignment to evolutionarily related gene groups was established by phylogenetic analysis. Sequences of SOXE, FGF9/16/20, and TGF-β groups of other vertebrates were retrieved from the NCBI protein database and ENSEMBL. Multiple alignments were performed with ClustalW2 using default parameters. Phylogenetic trees were obtained using Bayesian Inference (BI) and Maximum Parsimony (MP) methods. BI analysis was performed with MrBayes 3.1.2 [59] by applying the amino acid model of Dayhoff et al. [60] to the SOXE and TGF-β groups and the one by Jones et al. [61] to the FGF9/16/20 group. Parameters were set to 1,000,000 generations, sampling every 100; burn-in was set at 2,500 and stationarity was defined when the average standard deviation of split frequencies reached a value<0.009.

MP analyses were performed with PAUP [62] by applying heuristic search with tree bisection-reconnection (TBR) branch swapping and random stepwise additions with 100 replications; 1,000 bootstrap replicates were calculated. Only minimal trees were retained. The outgroup, accession numbers, and constant, parsimony informative, and parsimony non-informative sites are reported in the legend to each phylogenetic tree.

Conserved syntenic blocks were inferred from ENSEMBL annotation of putative *CYP11B* (Figure S2), *DMRT1*, *FGF9*, *FGF16*, and *FGF20* flanking regions from some sequenced vertebrate genomes. Gene sizes and distances were calculated on the basis of the annotated coordinates of each element. Scaffolds containing *FGF9* and flanking genes (*EFHA1* and *ZDHHC20*) conserved in tetrapods were identified by homology through tBLASTn on *L. chalumnae* WGS data.

## Results

GO analyses of ‘sex determination’ and ‘sex differentiation’ term annotations of the *L. menadoensis* transcriptome were conducted and the results compared to selected vertebrate genomes (Tables S1 and S2); 25 contigs were identified as orthologues of a GO0007530 (sex determination) annotation, and 297 contigs were orthologues of the GO0007544 (sex differentiation) annotation.

In this study we examined 33 genes with substantial evidence of involvement in sex determination and differentiation (Supplementary notes). CDSs were retrieved from the *L. chalumnae* genome and the *L. menadoensis* testis and liver transcriptomes (Tables 1 and 2) and their expression levels assessed. The putative orthology status of closely related genes was confirmed by tree topologies obtained by phylogenetic analysis. Furthermore the instances of micro-synteny conservation described in other vertebrates for *DMRT1* [36] and *FGF9/16/20* [63], [64] were analysed in the two coelacanths.

**Table 1:** Male sex-determining/differentiation gene inventory.

	Transcript in *L. menadoensis*			Gene location in *L. chalumnae*				Transcript prediction in *L. chalumnae*		
Gene	Accession	Length	CDS	Scaffold	N° exons^3^	Divergence^4^	Ka/Ks	ENSEMBL accession	Length	CDS
*AMH*	HF562302	1312^1^	1312^2^	JH126742	>5	0.046	0.000	ENSLACT00000009808	1689	1689^2^
	HF562303	1039^1^	670							
*AMHR2*	HF562304	693	693^2^	JH126659	>9	0.289	0.343	ENSLACT00000020587	921	921^2^
*AR*	HF562305	2590	2133^2^	JH126641	>8	0.165	0.000	ENSLACT00000017177	2235	1239^2^
*CYP11B*	-	-	-	JH127279	-	-	-	ENSLACT00000015536	1422	474^2^
*DAX1*	HF562306	966	786	JH128268	2	0.207	0.309	ENSLACT00000007979	786	786
*DHH*	HF562307	926	926^2^	JH126563	2	0.540	0.649	ENSLACT00000021749	1275	1275
*DMRT1*	HF562308	2244	998^2^	JH127237	5	0.134	0.000	ENSLACT00000015034	798	798^2^
*DMRT3*	-	-	-	JH127237	-	-	-	ENSLACT00000013757	1455	1455
*DMRT6*	HF562309	3121	957	JH130928	4	0.129	NA	ENSLACT00000003773	798	798^2^
*FGF9*	-	-	-	JH128123	-	-	-	Manually identified		
*FGF20*	HF562310	370	370^2^	JH127134	3	0.270	NA	ENSLACT00000014939	627	627
*GATA-4*	HF562311	1655	1200	JH128461	>6	0.064	NA	ENSLACT00000007000	1209	1209
*GSDF*	HF562312	1258	693	JH127632^5^	>3	0.826	0.470	-	-	-
*PDGFα*	HF562313	968	594	JH126909	6	0.000	NA	ENSLACT00000025036	1892	594
*PDGFβ*	HF562314	664	483^2^	JH128946	>4	0.000	NA	ENSLACT00000002931	630	630
*PDGFRα*	HF562315	823	823^2^	JH128279	>7	0.243	NA	ENSLACT00000010417	3285	3285^2^
*PDGFRβ*	HF562316	995^1^	995^2^	JH126585	>17	0.195	0.954	ENSLACT00000016664	3312	3312
	HF562317	1055^1^	1055^2^							
*SF-1*	HF562318	1686	1401	JH126572^6^	7	0.000	NA	ENSLACT00000021404	591	591^2^
*SOX8*	HF562319	735	735^2^	JH126713	>3	0.000	NA	ENSLACT00000019016	1434	1410
*SOX9*	HF562320	3306	1428	JH126581	3	0.185	0.000	ENSLACT00000021484	1908	1428
*SOX10*	HF562321	1403	1353^2^	JH127309	3	0.359	0.199	ENSLACT00000005034	4571	1356
*SRD5A1*	HF562322	3664	786	JH129903	5	0.164	NA	ENSLACT00000002047	6104	684
*SRD5A2*	HF562323	2711	765	JH126700	5	0.112	NA	ENSLACT00000025936	2918	765
*SRD5A3*	HF562324	1244	945	JH127256	5	0.000	NA	ENSLACT00000014423	2733	945
*WT1*	HF562325	2260	1257	JH126652	9	0.134	0.000	ENSLACT00000018732	1260	1257

1 Fragmented contig. ^2^Partial CDS. ^3^Number of exons from the alignment of *L. menadoensis* transcripts to the *L. chalumnae* genome. Where the transcript carries only a partial CDS, the number of exon is partial. ^4^Divergence between the two coelacanth sequences calculated as p-distance x100. ^5^The *L. chalumnae GSDF* gene is split between scaffold JH127632 and contig AFYH01270444. ^6^ The *L. chalumnae SF-1* gene is split between scaffold JH126572 and contig AFYH01271535.

**Table 2:** Female sex-determining/differentiation gene inventory.

	*L. menadoensis* transcript			Gene location in *L. chalumnae*				Transcript prediction in *L. chalumnae*		
Gene	Accession	Length	CDS	Scaffold	N° exons^3^	Divergence^4^	Ka/Ks	ENSEMBL accession	Length	CDS
*Aromatase*	-	-	-	JH127307	-	-	-	ENSLACT00000010703	1329	1329^2^
*CTNNB1*	HF562326	3325	2346	JH127054	15	0.702	0.000	ENSLACT00000017335	3458	2346
*ERα*	HF562327	2002	1541^2^	JH129227^5^	>8	0.352	NA	ENSLACT00000005056	396	396^2^
*ERβ*	HF562328	3184	1689	JH126564	9	0.159	0.000	ENSLACT00000019235	2465	1689
*FOXL2*	-	-	-	JH127245	-	-	-	ENSLACT00000012991	915	915
*FST*	HF562329	2381	1044	JH127291	6	0.221	0.000	ENSLACT00000014112	2027	1032^2^
*RSPO-1*	HF562330	474^1^	425^2^	JH126592	>5	0.626	NA	ENSLACT00000019383	747	747^2^
	HF562331	485^1^	269^2^							
*WNT4*	-	-	-	JH126950	-	-	-	ENSLACT00000017139	1068	1068

1 Fragmented contig. ^2^Partial CDS. ^3^Number of exons from the alignment of *L. menadoensis* transcripts to the *L. chalumnae* genome. Where the transcript carries only a partial CDS, the exon number is partial. ^4^Divergence between the two coelacanth sequences calculated as p-distance x100. ^5^The *ERα* gene in the *L. chalumnae* genome is split among scaffolds JH129227, JH129408, JH129637, and JH133026.

To establish whether the sequence information from *L. menadoensis* and *L. chalumnae* could be combined, their genetic distance was determined by comparing the transcripts of the former to the genomic sequences of the latter. The distance, calculated over all matching sequences, ranges between 0% and 0.826%, divergences being due mainly to mutations, insertions or deletions in untranslated regions (UTRs). Point mutations affecting the transcript coding region are predominantly synonymous (Tables 1 and 2). The synonymous distance calculated over the whole gene set was 0.0019 (standard error 0.0005). These findings showed that the data of the two species can be pooled and investigated together.

### Genes in male sexual development

Twenty-five genes involved in male sexual development were analysed in *Latimeria*: 3 genes containing a double sex and mab-3 (DM) domain (*DMRT1*, *DMRT3*, and *DMRT6*); 3 genes belonging to the *SOXE* subfamily (*SOX8*, *SOX9*, and *SOX10*) of SRY-related HMG box transcription factors; other transcription factors including WT1, DAX1, GATA-4, DHH, SF-1; the signalling molecules PDGFα and β, GSDF, AMH, FGF9, and FGF20; 4 receptors comprising AR, AMHR2, and PDGFRα and β; and the steroidogenic enzymes SRD5A1, SRD5A2, SRD5A3 and CYP11B (Table 1).

ENSEMBL prediction recovered 23 out of 25 genes in the *L. chalumnae* genome annotation. The two missing sequences were inferred manually from the genome assembly: one, *FGF9*, was identified by comparison with orthologous sequences of other species, and the other, *GSDF*, by aligning an *L. menadoensis* transcript to WGS contigs of *L. chalumnae*. Fifteen of the 23 predicted transcripts of *L. chalumnae* carried complete CDS whereas 8 were partial. The manually inferred *L. chalumnae FGF9* covers the complete CDS, whereas the *L. chalumnae GSDF* homologue is incomplete (about 75% of the CDS).

The testis and liver transcriptomes of *L. menadoensis* contain 22 transcripts. Half of the contigs carried a complete CDS, the other half were partial or fragmented. Transcripts of 3 genes, *FGF9*, *CYP11B*, and *DMRT3*, were not found in liver and testis (Table 1). The male sex development sequences of *L. menadoensis* and *L. chalumnae* are compared in Figure S1A.

**Figure 2:**
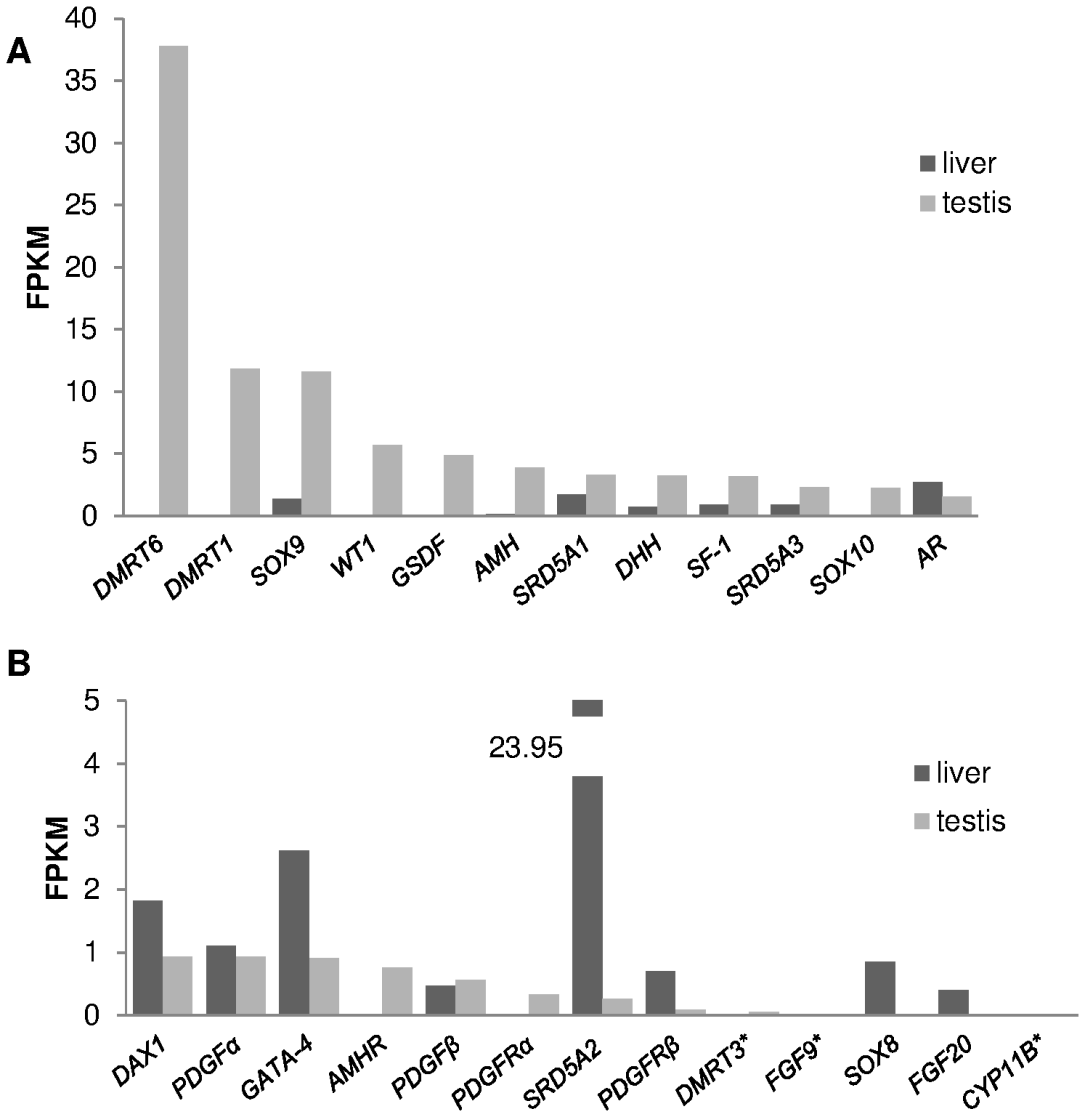
Expression of male development genes. Expression levels of male sex-determining/differentiation genes in *L. menadoensis* liver and testis transcriptomes. Values are expressed as FPKM (Fragments Per Kilobase of exon per Million sequenced fragments). A) genes highly expressed in testis; B) genes poorly expressed in testis. The expression levels of some housekeeping genes (not represented) were also analysed: *PGK* 96.95 (liver), 342.41 (testis); *RPS27a* 152.59 (liver), 128.43 (testis); *RPL19* 744.01 (liver), 64.89 (testis); *RPL11* 457.35 (liver), 282.59 (testis); *RPL32* 629.83 (liver), 373.75 (testis); *HSPCB* 507.99 (liver), 1213.75 (testis). Threshold value = 1. * Expression level assessed on *L. chalumnae* orthologue.

Since the expression of 13 male sex development transcripts in testis was<1 FPKM unit, they were considered as not being expressed above background. With the exception of *AR*, 11 genes (*DMRT6*, *DMRT1*, *SOX9*, *SOX10, WT1*, *GSDF*, *AMH*, *SRD5A1*, *SRD5A3, DHH*, and *SF-1*) were more expressed in testis than in liver, but only 3 (*DMRT1*, *DMRT6*, and *SOX9*) exhibited a differential expression with an FPKM difference>10. In liver 7 genes were expressed above background: *SOX9, SRD5A1, AR, DAX1, PDGFα, GATA-4*, and *SRD5A2* (Figures 2A and 2B).

*DMRT6* was the most highly expressed transcript among the 25 male sex development genes analysed (37.79 FPKM in testis, no expression in liver) and one of the 2,000 most abundant transcripts among the 61,000 plus contigs measured in testis.

**Figure 3:**
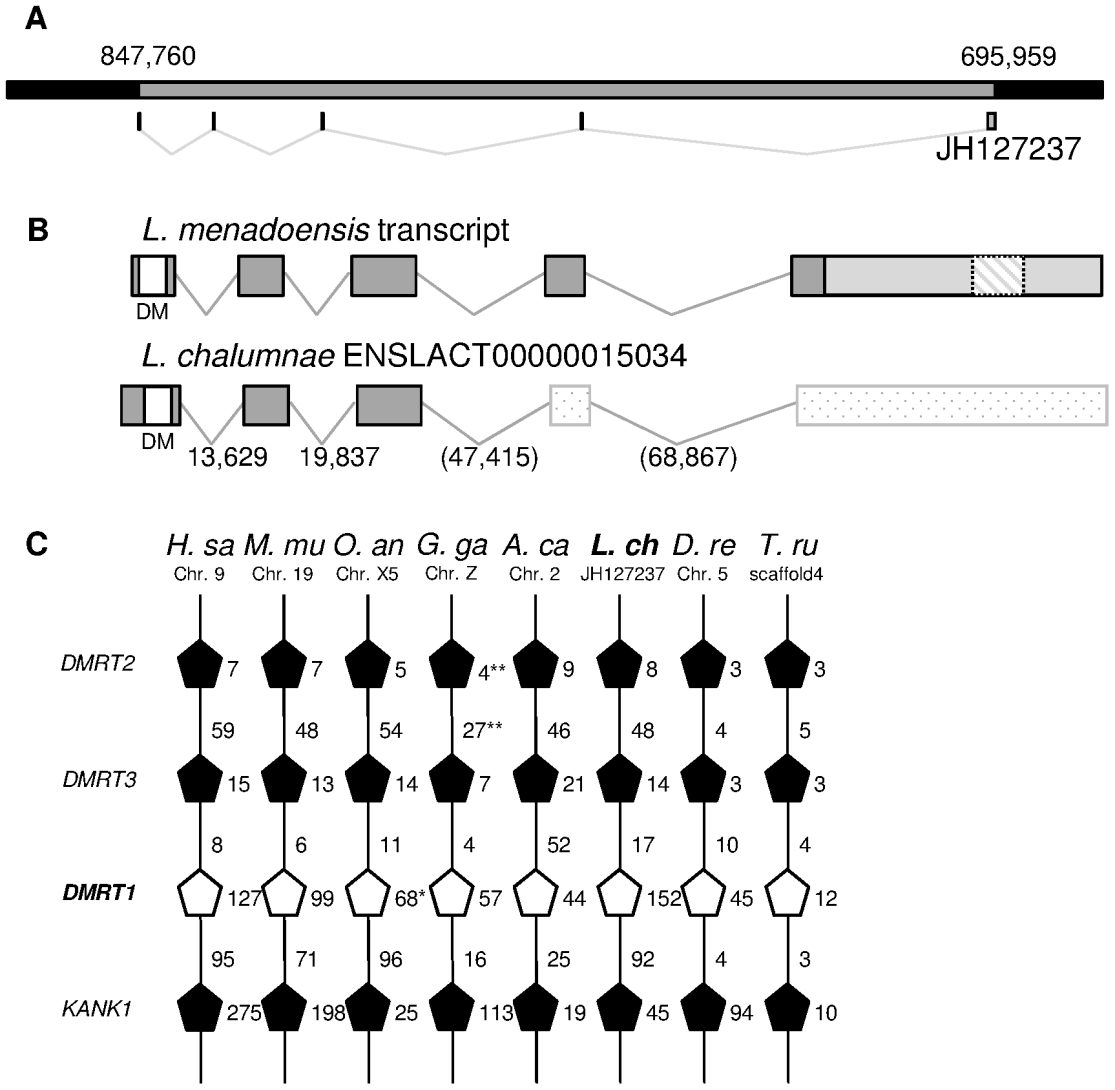
Conserved micro-synteny and structure of the *DMRT1* genomic locus and transcripts. A) Genomic representation of *DMRT1* on scaffold JH127237 of *L. chalumnae*. Grey box corresponds to gene. Small boxes and V signs represent the intron/exon map. B) Transcript representation of *DMRT1* in *L. menadoensis* and *L. chalumnae*. Boxes: exons; V signs: introns; white box: DM domain; light grey box: 3′UTR; dashed box: putative transposable element contained in the 3′UTR. Dotted boxes represent missing exons in the ENSEMBL transcript prediction. C) Micro-syntenic conservation of genomic blocks containing the *DMRT1* gene. White pentagons represent *DMRT1* genes. The pentagon tip points to the relative gene orientation. Numbers near the pentagons stand for gene size expressed as kb, numbers on lines represent intergene distance expressed as kb. ENSEMBL data: *H. sa* (*Homo sapiens*), *M. mu* (*Mus musculus*), *O. an* (*Ornithorhynchus anatinus*), *G. ga* (*Gallus gallus*), *A. ca* (*Anolis carolinensis*), *L. ch* (*Latimeria chalumnae*), *D. re* (*Danio rerio*), *T. ru* (*Takifugu rubripes*). *L. chalumnae DMRT1* position was clarified using the *L. menadoensis* transcript, by integrating the *L. chalumnae* ENSLACT00000015034 coordinates. *In *O. anatinus DMRT1* gene size was defined by comparison with other species. **Values obtained in *G. gallus* from the annotation of NC_006127.3 accession.

*DMRT1*, a major gene in male development, plays a key function in fish [65], [66], chickens [67], [68], and reptiles [69]. Alignment of *L. menadoensis* transcripts to the *L. chalumnae* genome (Figure 3A) identified 5 exons which exceeded the ENSEMBL predicted transcript by 1,572 bp at the 3′ end (Figure 3B). The DM domain is encoded in the first annotated exon. The long 3′UTR harbours a 320-bp region containing a low-copy interspersed repeat.

The size of the *DMRT1* gene in the *L. chalumnae* genome is>152 kb (Figure 3A), close to the 127 kb gene of *H. sapiens* (ENSEMBL annotation) but spanning a much longer range than the 3 kb gene of *Crocodylus palustris* [70], the 45 kb gene of *D. rerio* [71], and 53–58 kb gene of *G. gallus* ([72], ENSEMBL). Moreover the lack of a 5′ UTR (Figure 3B), which in other fish is transcribed in the so-called exon 0 [73], both in sequences from the transcriptome and the ENSEMBL prediction, suggests the existence of another exon (which would further extend the genomic locus).

Brunner and colleagues [36] previously reported that the gene order around the *DMRT1* gene, involving two other DM domain genes, *DMRT2* and *DMRT3*, and the gene *KANK1* (*KIAA0172*), was strictly conserved. A similar micro-synteny conservation was also noted in the *L. chalumnae* genome when the genomic scaffold JH127237 (1,057,921 bp), from position 608,000 to 941,000, was compared to other vertebrate chromosomes (Figure 3C). Interestingly, this region is linked to the Z gonosome in *G. gallus* (where *DMRT1* is pivotal in male development) and to the X5 gonosome in *Ornithorhynchus anatinus*, whereas in other species of the actinopterygian and sarcopterygian lineages it is located on an autosome. To date it has been impossible to identify sex chromosomes in the *Latimeria* karyotype [74] or to relate the scaffold containing *DMRT1* to a definite chromosome.

*DMRT1* is the second most abundantly expressed gene in testis (11.84 FPKM units) and among the 10% most abundantly expressed transcripts (Figure 2A) of those analysed.

**Figure 4:**
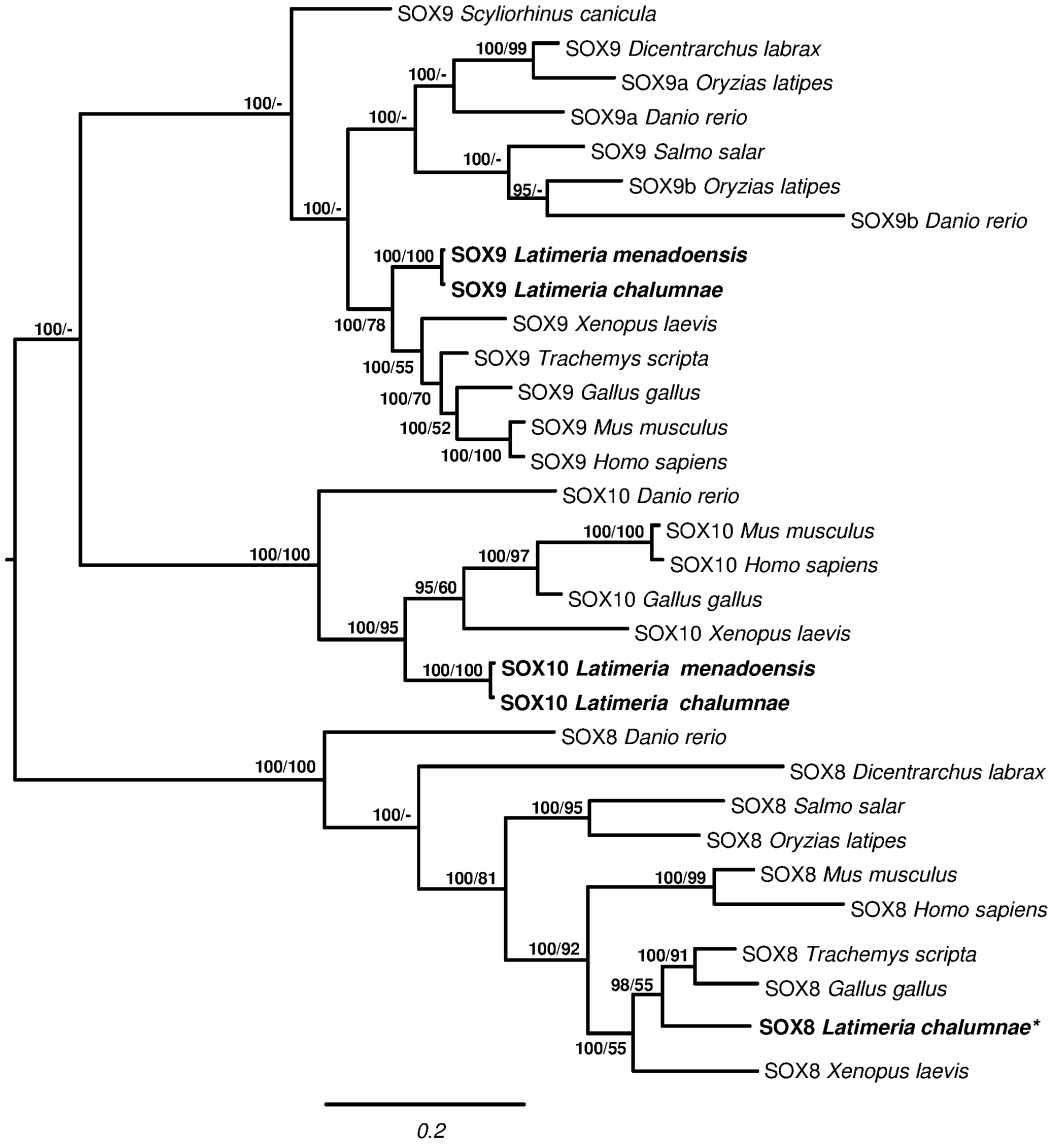
Phylogenetic tree of SOX8, SOX9, and SOX10. Phylogenetic analyses of vertebrate SOXE amino acid sequences. Midpoint rooting. Total characters: 592, constant: 164, parsimony non-informative: 77, parsimony informative: 351. Numbers close to nodes represent posterior probability in Bayesian Inference/bootstrap percentage in Maximum Parsimony. *Danio rerio* (SOX8: AAX73357.1; SOX9a: NP_571718.1; SOX9b: NP_571719.1; SOX10: AAK84872.1); *Dicentrarchus labrax* (SOX8: CBN81184.1; SOX9: CBN81190.1); *Gallus gallus* (SOX8: AAF73917.1; SOX9: BAA25296.1; SOX10: AAD38050.2); *Homo sapiens* (SOX8: AAH31797.1; SOX9: CAA86598.1; SOX10: CAG30470.1); *Latimeria chalumnae* (SOX8: ENSLACP00000018883; SOX9: ENSLACP00000021343; SOX10: ENSLACP00000004990); *Latimeria menadoensis* (SOX9, SOX10: this study); *Mus musculus* (SOX8: AAF35837.1; SOX9: AAH23953.1; SOX10: NP_035567.1); *Oryzias latipes* (SOX8: NP_001158342.1; SOX9a: AAX62152.1; SOX9b: AAX62151.1); *Salmo salar* (SOX8: ABC24688.1; SOX9: ACN10975.1); *Scyliorhinus canicula* (SOX9: ABY71239.1); *Trachemys scripta* (SOX8: AAP59791.1; SOX9: ACG70782.1; SOX10: ENSLACP00000004990); *Xenopus laevis* (SOX8: AAI69525.1; SOX9: NP_001084276; SOX10: NP_001082358.1). *Only a partial *SOX8* sequence, perfectly matching the ENSEMBL prediction of the *L. chalumnae SOX8* gene, was retrieved in the transcriptome assembly of *L. menadoensis*.

SOX9 is a transcription factor activating *AMH*; together with DMRT1 it inhibits *WNT4* and *FOXL2*. In mammals it is activated by another SOX family protein, SRY, whereas in other vertebrates it is mainly regulated by SF-1 and DMRT1; together with SOX8 and SOX10 it belongs to SOX protein subgroup E. Phylogenetic analysis (Figure 4) of SOX E proteins from several vertebrates yielded a tree topology with 3 major clades corresponding to the 3 genes. In the SOX9 and SOX10 clades *Latimeria* sequences comprise a sister group of tetrapods, while the relationship of the *Latimeria* SOX8 was not clearly resolved given its phylogenetic position. *SOX9* and *SOX10* were more strongly expressed in testis than in liver (Figure 2A; FPKM: 11.60 and 1.38 for *SOX9*, FPKM: 2.25 and 0.04 for *SOX10*), whereas *SOX8* expression was scanty in *L. menadoensis* liver (Figure 2B).

**Figure 5:**
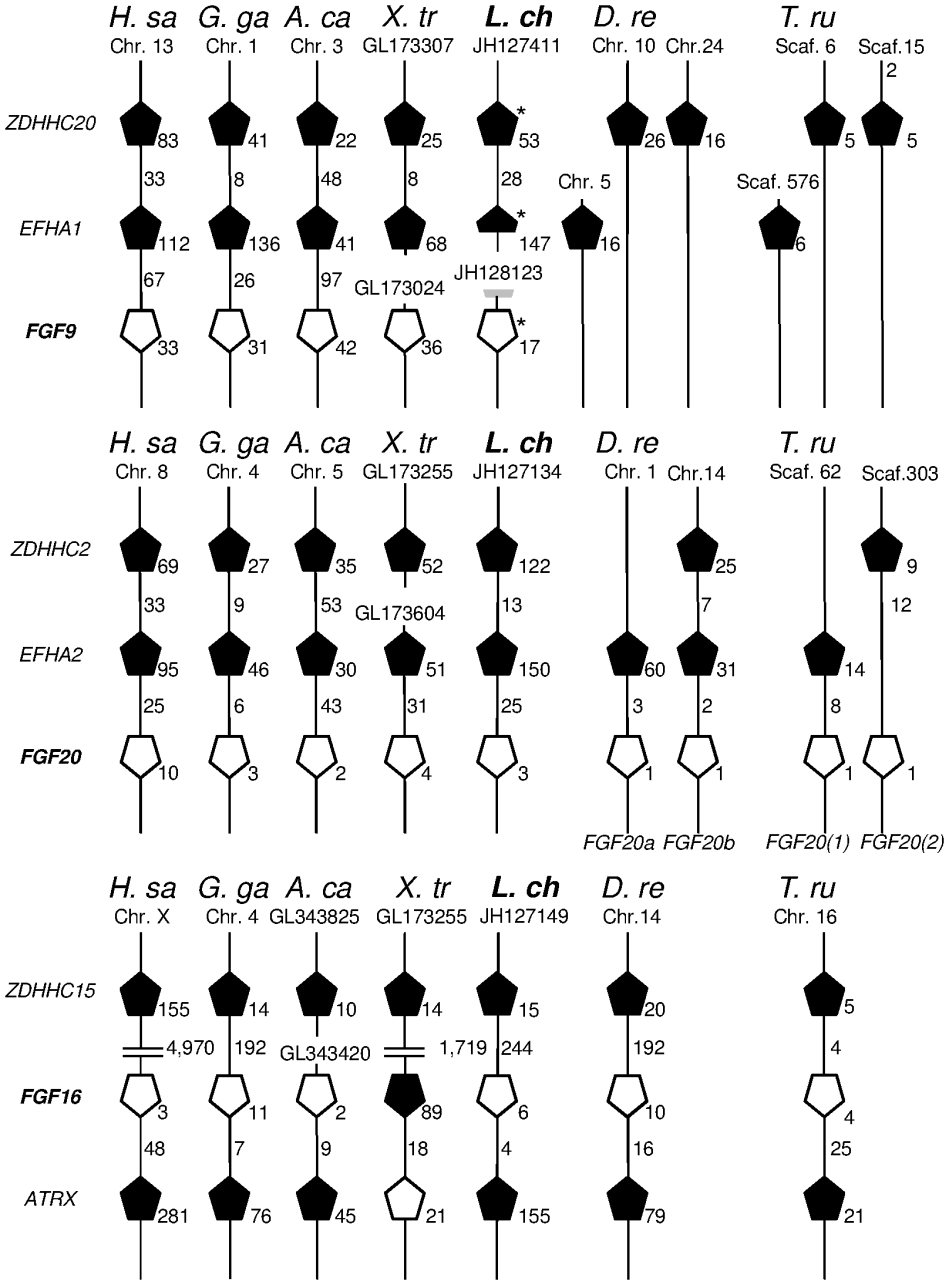
Analysis of micro-syntenic conservation in *FGF9*, *FGF16* and *FGF20* blocks. Micro-syntenic conservation of genomic regions containing the *FGF9*, *FGF20* and *FGF16* genes. White pentagons represent *FGF* genes. The pentagon tip points to the relative gene orientation. The grey mark on the top third of the figure indicates a *EFHA1* putative sequence of *Latimeria chalumnae*. Numbers near pentagons stand for gene size expressed as kb, numbers on lines represent intergene distance expressed as kb. ENSEMBL data: *H. sa* (*Homo sapiens*), *G. ga* (*Gallus gallus*), *A. ca* (*Anolis carolinensis*), *X. tr* (*Xenopus tropicalis*), *L. ch* (*Latimeria chalumnae*), *D. re* (*Danio rerio*), *T. ru* (*Takifugu rubripes*). Syntenic blocks for *FGF20* in *L. chalumnae* and *X. tropicalis*, and *FGF16* in *A. carolinensis* are split between two different scaffolds. The *ZDHHC15* genes belonging to the syntenic block of *FGF16* in *H. sapiens* and *X. tropicalis* lie on the same chromosome or scaffold, but are far removed from the genomic locus of *FGF16* and *ATRX*. *Genes missing in the ENSEMBL prediction.

In mammals FGF9 has an important function in male development, creating a positive feedback cycle with *SOX9* and inhibiting the WNT4 pathway in testis [75]. It has not yet been detected in teleosts and seems to be replaced by FGF20b [63], [64] in sexual development. Interestingly, we found an *FGF9*-like sequence in *L. chalumnae*. To confirm the orthology relationships of the putative *Latimeria FGF9*, *FGF16*, and *FGF20*, sequence comparisons were performed and the conserved synteny arrangements of the flanking regions investigated (Figure 5). In tetrapods the two blocks harbouring *FGF9* or *FGF20* are characterized by an *EFHA* and a *ZDHHC* gene upstream the *FGF* genes. Extensive gene-deserted regions are found downstream *FGF9, 16* and *20*. In teleosts (where *FGF9* is absent) the other genes forming the micro-syntenic cluster are distributed on different chromosomes. In *L. chalumnae* the *FGF9* cluster is split between two scaffolds whose co-localization on the same chromosome cannot as yet be confirmed. However, the proximity of a putative *EFHA1* coding fragment upstream the 5′ end of *FGF9* suggests that the *Latimeria FGF9* follows the tetrapod pattern.

**Figure 6:**
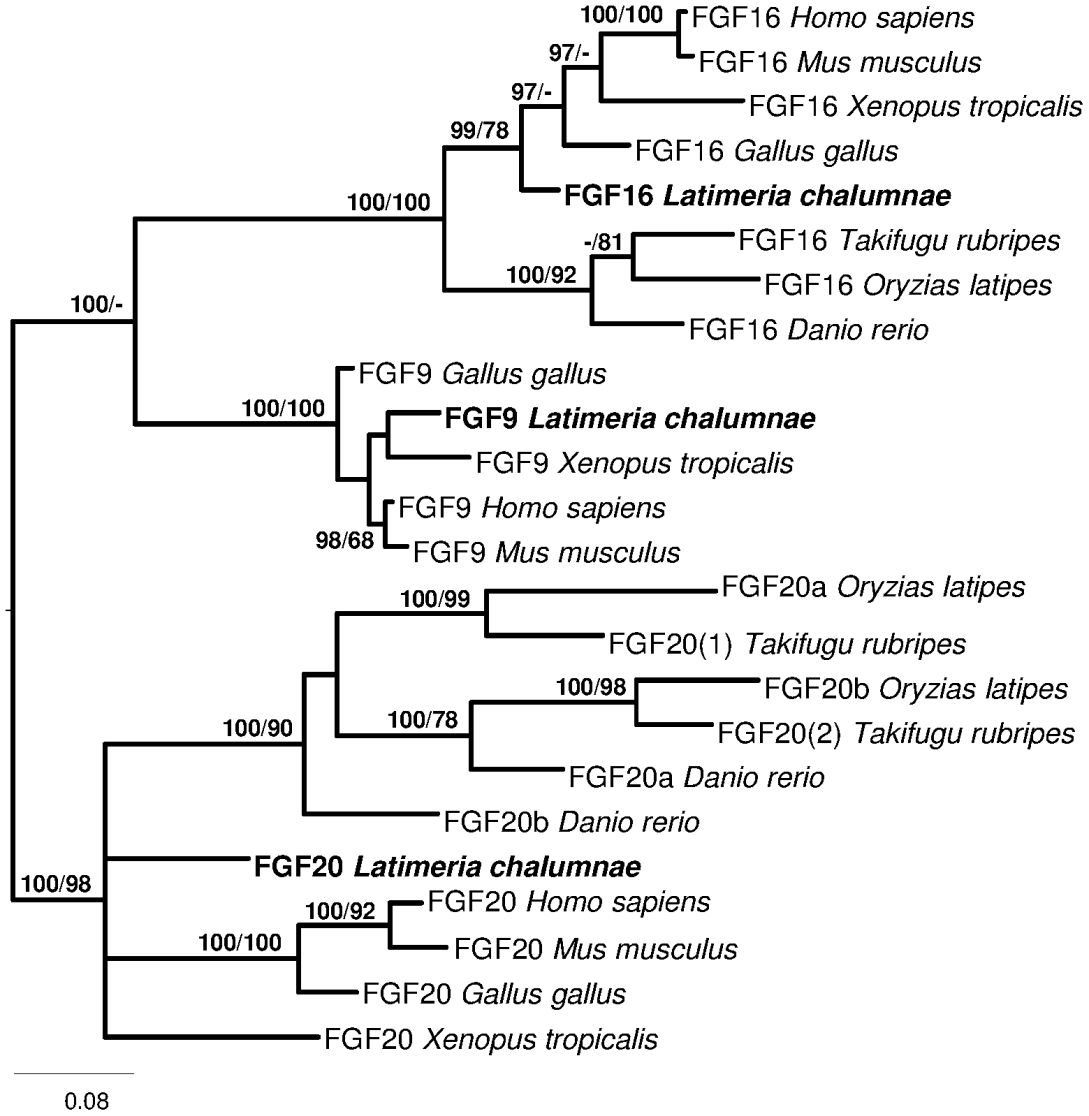
Phylogenetic tree of FGF9, FGF16, and FGF20. Phylogenetic analysis of amino acid sequences of the vertebrate FGF9/16/20. Midpoint rooting. Total characters: 237, constant: 91, parsimony non-informative: 35, parsimony informative: 111. Numbers close to nodes represent posterior probability in Bayesian Inference/bootstrap percentage in Maximum Parsimony. *Danio rerio* (FGF16: ENSDART00000061928; FGF20a: NP_001032180.1; FGF20b: NP_001034261.1); *Gallus gallus* (FGF9: NP_989730.1; FGF16; NP_001038115.1; FGF20: XP_426335.2); *Homo sapiens* (FGF9: NP_002001.1; FGF16: NP_003859.1; FGF20: NP_062825.1); *Latimeria chalumnae* (FGF9: manually inferred from JH128123; FGF16: ENSLACT00000011509; FGF20: ENSLACT00000014939); *Mus musculus* (FGF9: ADL60500.1; FGF16: BAB16405.1; FGF20: NP_085113.2); *Oryzias latipes* (FGF16: ENSORLT00000007651; FGF20a: ENSORLT00000012578; FGF20b: ENSORLT00000025767); *Takifugu rubripes* (FGF16: ENSTRUT00000021181; FGF20(1): ENSTRUT00000008788; FGF20(2): ENSTRUT00000039390); *Xenopus tropicalis* (FGF9: XP_002938621.1; FGF16: ENSXETT00000009790; FGF20: NP_001137399.1). *Latimeria menadoensis* is missing in this analysis because *FGF9* and *FGF20* are poorly or not expressed in the transcriptomes.

Phylogenetic analysis of the FGF9/16/20 group (Figure 6) uncovered three major clades corresponding to the 3 genes. The exact position of *L. chalumnae* FGF20 sequence is unresolved; like the *X*. *laevis* orthologue it is paraphyletic to teleosts and tetrapods. As expected, the coelacanth FGF16 sequence is basal to the tetrapods. However, the position of the *Latimeria* FGF9, albeit firmly nested within the FGF9 tetrapod clade, does not reflect its phylogenetic position in the taxonomic group.

Unexpectedly, neither *FGF9* nor *FGF20* expression was found in *L. menadoensis* testis.

**Figure 7:**
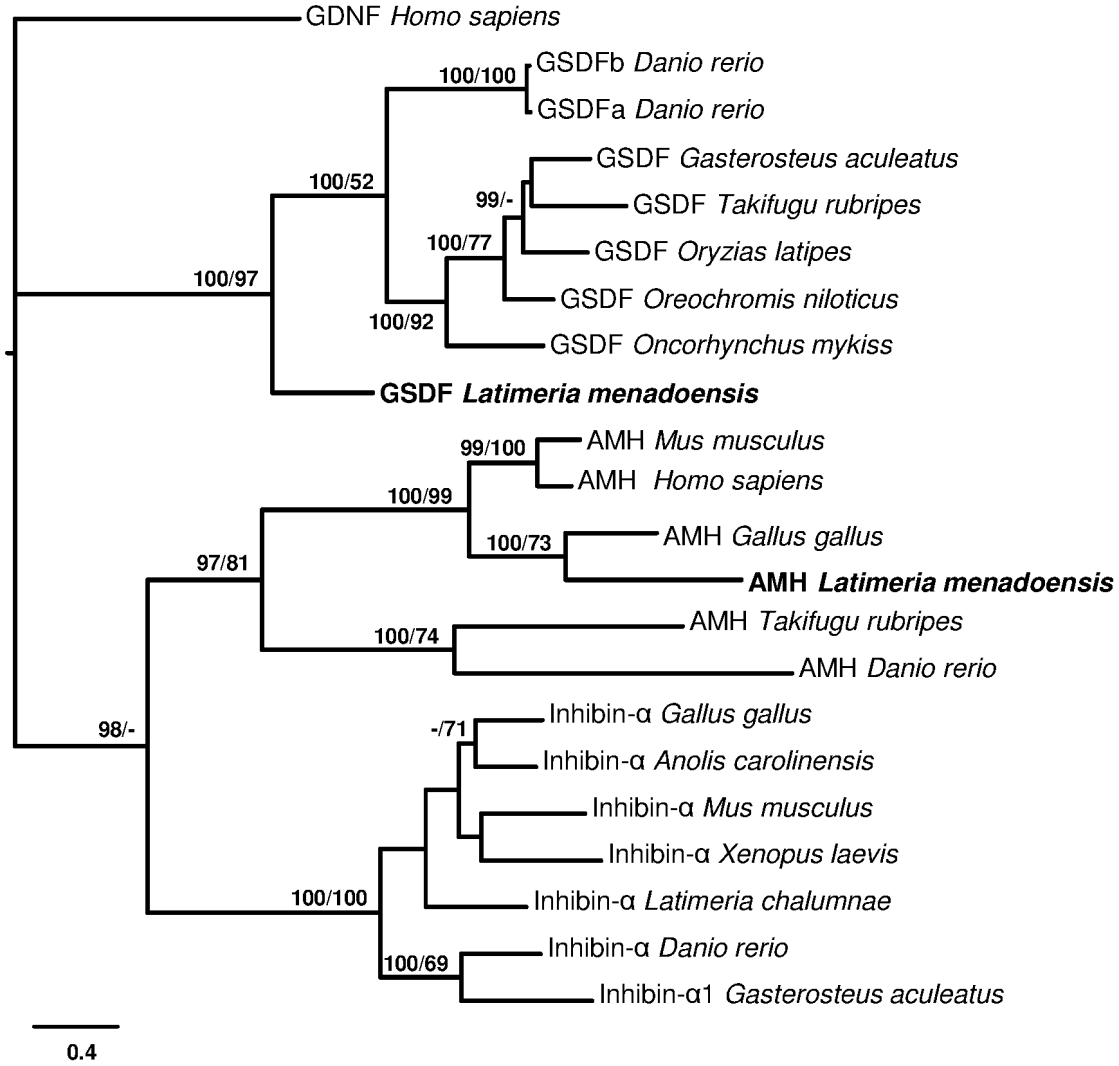
Phylogenetic tree of GSDF, AMH, and inhibin-α. Phylogenetic analysis of amino acid sequences of vertebrate GSDF, inhibin-α and AMH. Total characters: 849, constant: 84, parsimony non-informative: 225, parsimony informative: 540. Outgroup: human glial-derived nerve growth factor (GDNF). Numbers close to nodes represent posterior probabilities in Bayesian Inference/bootstrap percentage in Maximum Parsimony. *Anolis carolinensis* (inhibin-α: ENSACAT00000014331); *Danio rerio* (GSDFa: AEL99890.1; GSDFb: AEL99889.1; AMH: NP_001007780.1; inhibin-α: ENSDART00000057348); *Gallus gallus* (AMH: NP_990361.1; inhibin-α: NP_001026428.1); *Gasterosteus aculeatus* (GSDF: ENSGACT00000021595; inhibin-α: ENSGACT00000018909); *Homo sapiens* (AMH AAC25614.1; GDNF: NP_000505.1); *Latimeria chalumnae* (inhibin-α: ENSLACT00000017535); *Latimeria menadoensis* (GSDF, AMH this study); *Mus musculus* (AMH: AAI50478.1; inhibin-α: AAH56627.1); *Oreochromis niloticus* (GSDF: BAJ78985.1); *Oryzias latipes* (GSDF: NP_001171213.1); *Oncorhynchus mykiss* (GSDF: ABF48201.1); *Takifugu rubripes* (GSDF: ENSTRUT00000036269; AMH: ENSTRUT00000045919); *Xenopus laevis* (inhibin-α: NP_001106349.1). The reliability of *L. menadoensis* CDSs is supported by the same sequence resulting from application of two different assembly procedures.

**Figure 8:**
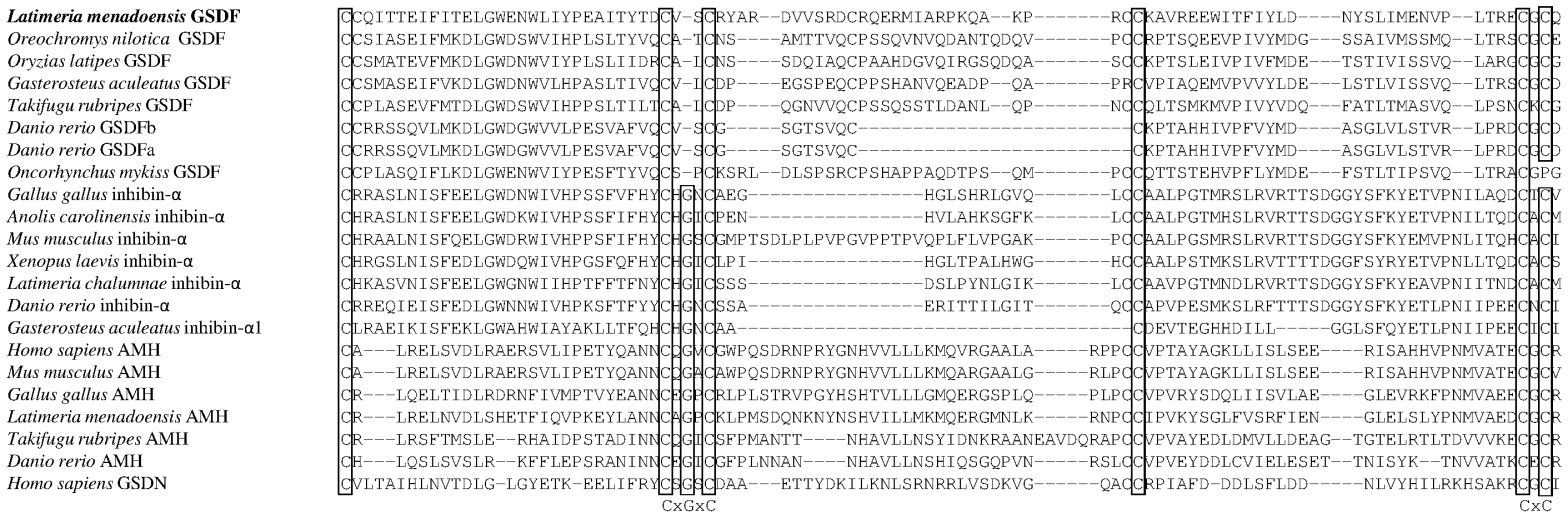
Multiple alignment of the TGF-β domain in GSDF, AMH, and inhibin-α. Conserved amino acids of the cysteine knot are boxed. *Anolis carolinensis* (inhibin-α: ENSACAT00000014331); *Danio rerio* (GSDFa: AEL99890.1, GSDFb: AEL99889.1; AMH: NP_001007780.1; inhibin-α: ENSDART00000057348); *Gallus gallus* (AMH: NP_990361.1; inhibin-α: NP_001026428.1); *Gasterosteus aculeatus* (GSDF: ENSGACT00000021595; inhibin-α: ENSGACT00000018909); *Homo sapiens* (AMH: AAC25614.1; GDNF: NP_000505.1); *Latimeria chalumnae* (inhibin-α: ENSLACT00000017535); *Latimeria menadoensis* (this study); *Mus musculus* (AMH: AAI50478.1; inhibin-α: AAH56627.1); *Oreochromis niloticus* (GSDF: BAJ78985.1); *Oryzias latipes* (GSDF: NP_001171213.1); *Oncorhynchus mykiss* (GSDF: ABF48201.1); *Takifugu rubripes* (GSDF: ENSTRUT00000036269; AMH: ENSTRUT00000045919); *Xenopus laevis* (inhibin-α: NP_001106349.1). The reliability of *L. menadoensis* CDSs is supported by the same sequence resulting from application of two different assembly procedures.

*GSDF*, a recently described gene that appears to be critically involved in the development of male teleosts [29], [39], [40], [45], has not been found in tetrapods and no sarcopterygian homologue has yet been described. However, BLAST analysis of teleost *GSDF* in the *L. menadoensis* transcript database suggested a putative *GSDF* gene, whose identity was confirmed by BLASTx analysis. Despite low similarity values (29% identity, 49% positive matching with *Oncorhynchus mykiss* GSDF NP_001118051.1, and 28% identity and 50% positive matching with *O. latipes* GSDF NP_001171213.1), BI and MP analyses reliably assigned the sequence to the teleost GSDF clade (Figure 7). Besides GSDF the phylogenetic analysis included two other proteins of the TGF-β family, AMH and inhibin-α, selected for their close relationships to GSDF [39]. A multiple amino acid alignment of the conserved TGF-β domain of the 3 genes disclosed that the *L. menadoensis* GSDF is a sister group of teleost GSDFs, with a posterior probability of 100 in BI analysis and a bootstrap value of 97 in the MP tree (Figure 8). The lack of a glycine, a diagnostic amino acid not found in the GSDF protein [45], in a cysteine knot further confirms the inclusion of the *L. menadoensis* sequence in the GSDF group, the first homologue to be described in the sarcopterygian lineage.

BLAST analysis of *L. menadoensis GSDF* on the *L. chalumnae* genome allowed identification of a genomic counterpart that was found partly on contig AFYH01270444 and partly on scaffold JH127632, with an intervening gap of 171 bp. The *L. menadoensis GSDF* is strongly expressed in testis but is not expressed in liver (Figure 2A).

### Genes in female sexual development

Eight female determining/differentiation genes were examined in the two coelacanths (Table 2): 3 genes belonging to the WNT signalling pathway (*WNT4*, *RSPO-1*, and *CTNNB1*), a transcription factor (FOXL2), two oestrogen receptors (ERα and ERβ), a steroidogenic enzyme (aromatase), and an activin-binding protein (FST).

ENSEMBL prediction recovered all 8 gene sequences in the *L. chalumnae* genome. Four transcripts (*ERβ*, *CTNNB1*, *WNT4*, and *FOXL2*) have a complete CDS; only two codons are missing at the 5′ end of *FST*; *RSPO-1* and *aromatase* are partial, whereas *ERα*, subdivided into 4 different scaffolds in the WGS, could be only partially identified.

Analysis of the *L. menadoensis* transcriptome yielded 3 complete CDS sequences (*CTNNB1*, *ERβ*, and *FST*) and 2 fragmented CDSs (*RSPO-1* and *ERα*), whereas 3 transcripts were missing (*FOXL2*, *WNT4*, and *aromatase*).

**Figure 9:**
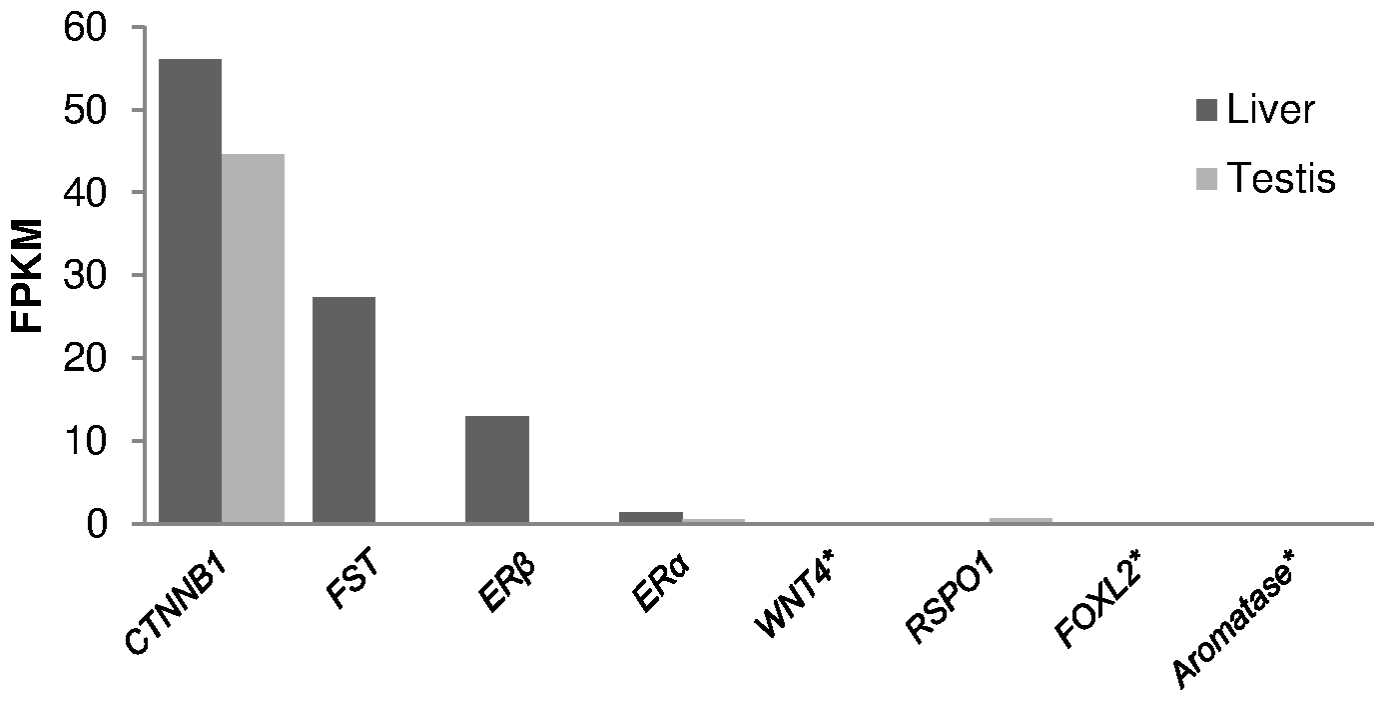
Expression of female development genes. Expression of female-determining/differentiation genes in *L. menadoensis* liver and testis transcriptomes. Expression levels are reported as FPKM (Fragments Per Kilobase of exon per Million sequenced fragments). The expression levels of some housekeeping genes were also analysed: *PGK* 96.95 (liver), 342.41 (testis); *RPS27a* 152.59 (liver), 128.43 (testis); *RPL19* 744.01 (liver) 64.89 (testis); *RPL11* 457.35 (liver), 282.59 (testis); *RPL32* 629.83 (liver), 373.75 (testis); *HSPCB* 507.99 (liver), 1213.75 (testis). Threshold value = 1. * Expression level assessed on *L. chalumnae* orthologue.

The female sex development sequences of *L. menadoensis* and *L. chalumnae* are compared in Figure S1B. Their values in *L. menadoensis* testis and liver are shown in Figure 9. As expected, *WNT4*, *FOXL2* and *aromatase* — held to be responsible for female development and pathway maintenance — were not expressed in testis. *CTNNB1*, *FST*, and *ERβ* were a strongly expressed in liver (56.08, 27.33, 12.93 FPKM, respectively); the expression of *FST* and *CTNNB1* was expected, because their expression is ubiquitous [76]. Finally, *ERβ* liver expression in the *L. menadoensis* specimen, a male individual, was unexpected.

## Discussion

In this study a set of 33 genes held to be critically involved in sexual development were isolated and characterized for the first time in coelacanths. Comparison of the gene sequences of the two *Latimeria* species confirmed the very slow rate of gene evolution that has recently been documented in *HOX* genes [77], although the latter genes are known to evolve particularly slowly. The 33 genes examined belong to a range of different families, thus providing valuable information.

Interpretation of our data is of course limited by the fact that they come from a single adult individual. However, given the importance of this living fossil in understanding tetrapod and fish evolution, and the exceptional opportunity provided by the availability of high-quality RNA from a specimen of an endangered species, we nonetheless cautiously draw some conclusions.

Ka/Ks analysis indicated that no gene in the set studied here is under positive selection in coelacanths. A totally unexpected finding was the very high *DMRT6* expression in testis, which was actually the most abundant male-specific transcript. To date the gene had only been found in amniotes and is not annotated in *Xenopus* and all fish genomes. This phylogenetic pattern could be explained by its being a newly arisen paralogue of the *DMRT* family at the base of amniote vertebrates. Detection of a bona fide *DMRT6* homologue in *Latimeria* points to a much earlier origin of the gene and supports a possible origin from the 1R/2R whole genome duplication events that occurred in ancestral vertebrates [78] and a subsequent, repeated loss in the teleost fish and amphibian lineages and even in basal chordates. Since information on *DMRT6* expression is quite scanty the interpretation of some of these data is merely speculative. In mouse embryo it is expressed in the developing brain but not in the gonads [43]. In the human microarray database (https://www.genevestigator.com) it is highly expressed exclusively in ovary and testis, whereas studies of mouse organs have disclosed that only erythroblasts and oocytes show elevated expression. Whatever its original function, it is reasonable to assume that *DMRT6* was taken over by other members of the gene family, and that it has ceased to be required in those lineages where it is no longer extant. Its persistence in *Latimeria* may indicate an important function in male (and possibly female) development which, according to current knowledge, was then at least partially conserved in amniotes. Our findings suggest its being a putative novel gene in the gonad regulatory network.

The high *DMRT1* expression found in *Latimeria* testis and its lack of expression in liver is in line with its expression pattern and important role in testis development and in maintenance of the male gonad identity documented in vertebrates, from fish to mammals [65], [66]. In teleost fish adult testis *DMRT1* is found in germ cells, in somatic cell types or both [65]. Unfortunately, RNA-Seq transcriptome data provide no information on the cell type expressing *DMRT1* in coelacanth testis. In medaka a duplicated version of *DMRT1* on the Y chromosome, designated *dmrt1bY*, is the master male sex determining gene [26], [27]. Its major function appears to suppress germ cell proliferation at the critical sex-determining stage in males [79]. In adult testes it is dramatically downregulated [80], and its high expression suggests that only the autosomal *DMRT1* (*dmrt1a* in medaka) may function in mature testis. As in all the teleosts studied so far, a single *DMRT1* copy was found in *L. menadoensis*, suggesting that in coelacanths it may not serve a major role in primary sex determination, but may do so in testis differentiation and adult testis function.

Unlike all the other vertebrates studied [38], [43], [81]–[83], *DMRT3* is not expressed in *L. menadoensis* male gonad.

The *TGF-β* family member *GSDF* is an important gene in teleost fish gonad development and displays much higher expression in testis than in ovary [39], [40]. A duplicate of *GSDF* may actually have become the master male sex determination gene in *Oryzas luzonensis* [29]; there is strong evidence that in *O. latipes* the master male sex determining gene *dmrt1bY* upregulates *GSDF* and that upregulation correlates with early testis differentiation [45]. No *GSDF* homologue has yet been identified outside teleosts. Identification in our study of a bona fide *GSDF* sequence in *Latimeria* and its high expression in testis (which also points at its functional conservation) suggests that the gene arose already at the base of the fish lineage, but was later lost during tetrapod evolution. *GSDF* thus appears to be an ancestral male sex-determining gene. In the absence of functional data on *GSDF* function in fish, it remains unclear whether during tetrapod testis development another *TGF-β* family member may have taken over the function it exerted in teleosts and coelacanths.

The high expression in the *L. menadoensis* testis transcriptome of *SOX9*, *SOX10*, *WT1*, *AMH*, *DHH*, *SF-1* and *SDR5A1* and *3* (at least compared to liver), the low expression of *AMHR2*, and the absence of the female factors *FST*, *RSPO-1*, *WNT4*, *FOXL2*, *aromatase*, and *oestrogen receptor* transcripts are in line with their expression patterns documented in many vertebrate species and with their proposed function in sexual development.

In particular, the AMH/AMH-receptor system is of interest for *Latimeria* sexual development. In mammals and most likely in all tetrapods AMH induces Müllerian duct regression. Teleosts do not have Müllerian ducts, whereas lungfish and *Latimeria* possess oviducts that are homologous to those of tetrapods [84]. Despite the absence of Müllerian ducts, AMH/AMH-receptor system has an important function in the manifestation of gonadal sex in teleosts, because in medaka AMH signalling is crucial in regulating germ cell proliferation in early gonad differentiation [85]. Given that *AMH* and *AMH-receptor* are expressed in *L. menadoensis* adult testis, the AMH signalling system is present and probably active there, as in adult teleosts [86], [87], [88], whereas in mouse testis the system is downregulated before sexual maturity [89].

Several of the 33 genes tested, all of which are involved in sex determination and differentiation in other organisms, were found to be abundantly expressed in the liver transcriptome. The high *CTNNB1* levels were expected, due to the ubiquitous function of this signal transducer of the WNT pathway. High *FST* expression agrees with its expression in all vertebrates and with the finding that it is required for liver cell growth homeostasis in mice [90]. This non-gonadal function of the gene may be conserved in coelacanths. Similarly the transcription factor GATA-4, besides a role in gene regulation in testis development [91], is also involved in the control of a number of liver genes, explaining why transcripts of the coelacanth homologue were found in both tissues. In contrast to coelacanths, where *5α-reductase 2* is highly expressed in liver, the *5α-reductase 1* isoform is differentially regulated by androgens and glucocorticoids in rat liver, resulting in high expression in this tissue, while *5α-reductase 2* is preferentially expressed in gonads [92]. This may indicate lineage-specific sub-functionalization of the isozymes during evolution.

The absence of *SOX8* expression in *Latimeria* testis was unexpected. In other vertebrates, including teleost fish, it is readily detected in this organ, and in mammals it has been assigned an important function in the FGF9/SOX9 interaction loop to maintain Sertoli cell identity by acting redundantly to SOX9 [6], [93]. Such back-up function does not seem to be required in *Latimeria* testis maintenance, or may have been lost in the extant coelacanth lineage. In medaka SOX9 is required for germ cell proliferation and survival, but not for testis determination [94]. Together with the other *L. menadoensis* findings this may indicate that the sex-determining function was acquired later in tetrapod lineage, after the split of teleost and coelacanth lineages.

Intriguing data were found for *FGF9* and *20*, which together with *FGF16* constitute a gene subfamily of paracrine FGFs. The critical role of *FGF9* in mammalian testis development is well established and appears to be conserved in all tetrapods. On the other hand, the gene is not found in any teleost genome ([63], [64], ENSEMBL), unlike *FGF16* and *20* (the latter being duplicated due to the teleost genome duplication). In the amphioxus an *FGF* gene is basal to the three *FGFs* in tetrapods [95]. *FGF9* could thus be a later duplicate of either *FGF16* or *20*, and its role in testis development could be interpreted as an innovation arising in tetrapods. However, identification of *FGF9* in *Latimeria* supports an origin during the 1R/2R whole genome duplication events that took place in ancestral chordates and its loss in the lineage leading to teleosts. In the teleost *Oreochromis niloticus* (Tilapia) *FGF20b* and *FGF16* are both expressed in ovary, whereas only *FGF16* is (poorly) expressed in testis [64]. Together with the complete absence of *FGF9*, *FGF20* and *FGF16* expression in *L. menadoensis* liver and testis, this indicates that the function of FGF signalling in testis, in particular the central role of *FGF9*, was acquired later in tetrapod evolution.

Surprisingly, the *ERβ* gene was expressed in the liver of the male coelacanth. A previous study of the same individual had disclosed expression of the vitellogenin genes *vtgABI*, *II* and *III* [50]. Vitellogenins are yolk proteins physiologically expressed in the liver of reproductive females upon induction by oestrogens. Thus expression of vitellogenins and oestrogen receptor indicates the presence of oestrogens in this male specimen. They could derive from environment pollutants, as reported in a number of specimens from polluted waters; however this individual lived in Bunaken Marine Park in submarine caves at a depth of 100 to 200 m, i.e. in a relatively protected environment. Alternatively, *ERβ* expression could be the result of a pathological condition, of a hormone imbalance due to ageing, or of a physiological feature of coelacanths.

## Conclusions

Analysis of the coelacanth testis transcriptome, reported here for the first time, disclosed important new information on which genes involved in sexual development and testis differentiation in other organisms are present and expressed in this living fossil and on the evolution of this process in vertebrates. Interestingly, some genes that are generally considered critical for testis maintenance in all vertebrates, like *SOX8* or a fibroblast growth factor gene from the *FGF9/16/20* subfamily, do not play this role in *Latimeria*. This finding and the high *GSDF* expression found in the coelacanths make their transcript profile more similar to that of modern fish. In summary, the coelacanth testis transcriptome is expected to contribute further important information to reconstruct the ancestral tetrapod situation and indicates that evolutionary innovations for sexual development occurred already during the transition from water to land.

## Supporting Information

Figure S1 **Sequence pair comparison of male sex development genes.** Sequence pair comparison of male sex-determining/differentiation transcripts from the *L. menadoensis* transcriptome and *L. chalumnae* ENSEMBL predictions. Boxes represent CDSs. Lines represent UTRs. Dashed boxes represent a missing part in the CDS. Green lines/boxes represent an inaccurate gene prediction or a mismatch between *L. chalumnae* and *L. menadoensis* sequences. Scale dimension are preserved. B) Sequence pair comparison of female sex development genes. Sequence pair comparison of female sex-determining/differentiation transcripts from the *L. menadoensis* transcriptome and *L. chalumnae* ENSEMBL predictions. Boxes represent CDSs. Lines represent UTRs. Dashed boxes represent a missing part in the CDS. Green lines/boxes represent an inaccurate gene prediction or a mismatch between *L. chalumnae* and *L. menadoensis* sequences. Scale dimension are preserved.
(PDF)

Figure S2 **Micro-syntenic conservation of** ***CYP11B*** **.** Micro-syntenic conservation of genomic regions containing *CYP11B* genes. Black pentagons represent *CYP11B* genes. The pentagon tip points to the relative gene orientation. ENSEMBL data: *H. sa* (*Homo sapiens*), *M. mu* (*Mus musculus*), *B. ta* (*Bos taurus*), *L. ch* (*Latimeria chalumnae*), *D. re* (*Danio rerio*), *T. ru* (*Takifugu rubripes*).
(PDF)

Table S1 **Gene Ontology analysis of the “sex determination” term.**
(PDF)

Table S2 **Gene Ontology analysis of the “sex differentiation” term.**
(PDF)

## References

[1] Hayes TB (1998) Sex determination and primary sex differentiation in amphibian: genetic and developmental mechanisms. J Exp Zool 281: 373–399. 9662826

[2] Graves JAM, Peichel CL (2010) Are homologies in vertebrate sex determination due to shared ancestry or to limited options? Genome Biol 11: 205. 20441602 10.1186/gb-2010-11-4-205 PMC2884537

[3] McClelland K, Bowles J, Koopman P (2012) Male sex determination: insights into molecular mechanisms. Asian J Androl 14: 164–171. 22179516 10.1038/aja.2011.169 PMC3735148

[4] Uhlenhaut NH, Jakob S, Anlag K, Eisenberger T, Sekido R, et al (2009) Somatic sex reprogramming of adult ovaries to testes by FOXL2 ablation. Cell 139: 1130–1142. 20005806 10.1016/j.cell.2009.11.021

[5] Matson CK, Murphy MW, Sarver AL, Griswold MD, Bardwell VJ, et al (2011) *DMRT1* prevents female reprogramming in the postnatal mammalian testis. Nature 476: 101–104. 21775990 10.1038/nature10239 PMC3150961

[6] Herpin A, Schartl M (2011) Sex determination: switch and suppress. Curr Biol 21: R656–9. 21920296 10.1016/j.cub.2011.07.026

[7] Clinton M, Zhao D, Nandi S, McBride D (2012) Evidence for avian cell autonomous sex identity (CASI) and implications for the sex-determination process? Chromosome Res 20: 177–190. 22124858 10.1007/s10577-011-9257-9

[8] Zarkower D (2006) Somatic sex determination (February 10, 2006), in WormBook. Edited by The *C. elegans* Research Community.doi/10.1895/wormbook.1.84.1, http://www.wormbook.org. 10.1895/wormbook.1.84.1 PMC4781091 18050479

[9] Abinawanto, Shimada K, Yoshida K, Saito N (1996) Effects of aromatase inhibitor on sex differentiation and levels of P450_17 alpha_ and P450_arom_ messenger ribonucleic acid of gonads in chicken embryos. Gen Comp Endocrinol 102: 241–246. 8998968 10.1006/gcen.1996.0065

[10] Akazome Y, Mori T (1999) Evidence of sex reversal in the gonads of chicken embryos after oestrogen treatment as detected by expression of lutropin receptor. J Reprod Fertil 115: 9–14. 10341717 10.1530/jrf.0.1150009

[11] Baker PJ, Moore HD, Burgess AM, Mittwoch U (1993) Gonadal sex differentiation in embryos and neonates of the marsupial, *Monodelphis domestica*: arrest of testis development in postterm embryos. J Anat 182: 267–273. 8376201 PMC1259837

[12] Coveney D, Shaw G, Renfree MB (2001) Estrogen-induced gonadal sex reversal in the tammar wallaby. Biol Reprod 65: 613–621. 11466233 10.1095/biolreprod65.2.613

[13] Fadem BH (2000) Perinatal exposure to estradiol masculinizes aspects of sexually dimorphic behavior and morphology in gray short-tailed opossums (*Monodelphis domestica*). Horm Behav 37: 79–85. 10712860 10.1006/hbeh.1999.1561

[14] Guiguen Y, Baroiller JF, Ricordel MJ, Iseki K, Mcmeel OM, et al (1999) Involvement of estrogens in the process of sex differentiation in two fish species: the rainbow trout (*Oncorhynchus mykiss*) and a tilapia (*Oreochromis niloticus*). Mol Reprod Dev 54: 154–162. 10471475 10.1002/(SICI)1098-2795(199910)54:2<154::AID-MRD7>3.0.CO;2-5

[15] Kobayashi T, Kajiura-Kobayashi H, Nagahama Y (2003) Induction of XY sex reversal by estrogen involves altered gene expression in a teleost, tilapia. Cytogenet Genome Res 101: 289–294. 14684997 10.1159/000074351

[16] Mackenzie CA, Berrill M, Metcalfe C, Pauli BD (2003) Gonadal differentiation in frogs exposed to estrogenic and antiestrogenic compounds. Environ Toxicol Chem 22: 2466–2475. 14552012 10.1897/02-173

[17] Mittwoch U (1998) Phenotypic manifestations during the development of the dominant and default gonads in mammals and birds. J Exp Zool 281: 466–471. 9662833

[18] Pieau C, Dorizzi M (2004) Oestrogens and temperature-dependent sex determination in reptiles: all is in the gonads. J Endocrinol 181: 367–377. 15171684 10.1677/joe.0.1810367

[19] Renfree MB, Coveney D, Shaw G (2001) The influence of estrogen on the developing male marsupial. Reprod Fertil Dev 13: 231–240. 11800162 10.1071/rd00123

[20] Shaw G, Renfree MB, Short RV, WS O (1988) Experimental manipulation of sexual differentiation in wallaby pouch young treated with exogenous steroids. Development 104: 689–701. 3268410 10.1242/dev.104.4.689

[21] Ramsey M, Crews D (2009) Steroid signaling and temperature-dependent sex determination. Reviewing the evidence for early action of estrogen during ovarian determination in turtles. Semin Cell Dev Biol 20: 283–292. 18992835 10.1016/j.semcdb.2008.10.004 PMC2695493

[22] Schartl M (2004) Sex chromosome evolution in non-mammalian vertebrates. Curr Opin Genet Dev 14: 634–641. 15531158 10.1016/j.gde.2004.09.005

[23] Uller T, Helanterä H (2011) From the origin of sex-determining factors to the evolution of sex-determining systems. Q Rev Biol 86: 163–180. 21954700 10.1086/661118

[24] Biason-Lauber A (2010) Control of sex development. Best Pract Res Clin Endocrinol Metab 24: 163–186. 20541146 10.1016/j.beem.2009.12.002

[25] Kashimada K, Koopman P (2010) *Sry*: the master switch in mammalian sex determination. Development 137: 3921–3930. 21062860 10.1242/dev.048983

[26] Matsuda M, Nagahama Y, Shinomiya A, Sato T, Matsuda C, et al (2002) *DMY* is a Y-specific DM-domain gene required for male development in the medaka fish. Nature 417: 559–563. 12037570 10.1038/nature751

[27] Nanda I, Kondo M, Hornung U, Asakawa S, Winkler C, et al (2002) A duplicated copy of *DMRT1* in the sex-determining region of the Y chromosome of the medaka, *Oryzias latipes* . Proc Natl Acad Sci USA 99: 11778–11783. 12193652 10.1073/pnas.182314699 PMC129345

[28] Yoshimoto S, Okada E, Umemoto H, Tamura K, Uno Y, et al (2008) A W-linked DM-domain gene, DM-W, participates in primary ovary development in *Xenopus laevis* . Proc Natl Acad Sci USA 105: 2469–2474. 18268317 10.1073/pnas.0712244105 PMC2268160

[29] Myosho T, Otake H, Masuyama H, Matsuda M, Kuroki Y, et al (2012) Tracing the emergence of a novel sex-determining gene in medaka, *Oryzias luzonensis* . Genetics 191: 163–170. 22367037 10.1534/genetics.111.137497 PMC3338257

[30] Hattori RS, Murai Y, Oura M, Masuda S, Majhi SK, et al (2012) A Y-linked anti-Müllerian hormone duplication takes over a critical role in sex determination. Proc Natl Acad Sci USA 109: 2955–2959. 22323585 10.1073/pnas.1018392109 PMC3286941

[31] Kamiya T, Kai W, Tasumi S, Oka A, Matsunaga T, et al (2012) A Trans-Species Missense SNP in *Amhr2* Is Associated with Sex Determination in the Tiger Pufferfish, *Takifugu rubripes* (Fugu). PLoS Genet 8: e1002798. 22807687 10.1371/journal.pgen.1002798 PMC3395601

[32] Angelopoulou R, Lavranos G, Manolakou P (2012) Sex determination strategies in 2012: towards a common regulatory model? Reprod Biol Endocrinol 10: 13. 22357269 10.1186/1477-7827-10-13 PMC3311596

[33] Graves JAM (1995) The evolution of mammalian sex chromosomes and the origin of sex-determining genes. Philos Trans R Soc Lond B Biol Sci 350: 305–311. 8570696 10.1098/rstb.1995.0166

[34] Graves JAM (2008) Weird animal genomes and the evolution of vertebrate sex and sex chromosomes. Annu Rev Genet 42: 565–586. 18983263 10.1146/annurev.genet.42.110807.091714

[35] Borg B (1994) Androgens in teleost fishes. Comp Biochem Physiol Part C 109: 219–245.

[36] Brunner B, Hornung U, Shan Z, Nanda I, Kondo M, et al (2001) Genomic organization and expression of the doublesex-related gene cluster in vertebrates and detection of putative regulatory regions for *DMRT1* . Genomics 77: 8–17. 11543627 10.1006/geno.2001.6615

[37] Chardard D, Kuntz S, Chesnel A, Flament S (2003) Effects of androgens on sex differentiation of the urodele *Pleurodeles waltl* . J Exp Zoolog A Comp Exp Biol 296: 46–55. 10.1002/jez.a.10240 12589690

[38] El-Mogharbel N, Wakefield M, Deakin JE, Tsend-Ayush E, Grützner F, et al (2007) *DMRT* gene cluster analysis in the platypus: new insights into genomic organization and regulatory regions. Genomics 89: 10–21. 16962738 10.1016/j.ygeno.2006.07.017

[39] Gautier A, Le Gac F, Lareyre JJ (2011) The *gsdf* gene locus harbors evolutionary conserved and clustered genes preferentially expressed in fish previtellogenic oocytes. Gene 472: 7–17. 21047546 10.1016/j.gene.2010.10.014

[40] Gautier A, Sohm F, Joly JS, Le Gac F, Lareyre JJ (2011) The proximal promoter region of the zebrafish *gsdf* gene is sufficient to mimic the spatio-temporal expression pattern of the endogenous gene in Sertoli and granulosa cells. Biol Reprod 85: 1240–1251. 21816849 10.1095/biolreprod.111.091892

[41] Gnessi L, Emidi A, Jannini EA, Carosa E, Maroders M, et al (1995) Testicular development involves the spatiotemporal control of PDGFs and PDGF receptors gene expression and action. J Cell Biol 131: 1105–1121. 7490286 10.1083/jcb.131.4.1105 PMC2199998

[42] Gnessi L, Basciani S, Mariani S, Arizzi M, Spera G, et al (2000) Leydig cell loss and spermatogenic arrest in platelet-derived growth factor (PDGF)-A-deficient mice. J Cell Biol 149: 1019–1025. 10831606 10.1083/jcb.149.5.1019 PMC2174827

[43] Kim S, Kettlewell JR, Anderson RC, Bardwell VJ, Zarkower D (2003) Sexually dimorphic expression of multiple doublesex-related genes in the embryonic mouse gonad. Gene Expr Patterns 3: 77–82. 12609607 10.1016/s1567-133x(02)00071-6

[44] O'Donnell L, Stanton PG, Wreford NG, Robertson DM, McLachlan RI (1996) Inhibition of 5-alpha-reductase activity impairs the testosterone-dependent restoration of spermiogenesis in adult rats. Endocrinology 137: 2703–2710. 8770889 10.1210/endo.137.7.8770889

[45] Shibata Y, Paul-Prasanth B, Suzuki A, Usami T, Nakamoto M, et al (2010) Expression of gonadal soma derived factor (*GSDF*) is spatially and temporally correlated with early testicular differentiation in medaka. Gene Expr Patterns 10: 283–289. 20601164 10.1016/j.gep.2010.06.005

[46] Smith CA, McClive PJ, Hudson Q, Sinclair AH (2005) Male-specific cell migration into the developing gonad is a conserved process involving PDGF signaling. Dev Biol 284: 337–350. 16005453 10.1016/j.ydbio.2005.05.030

[47] Zaccanti F, Petrini S, Rubatta ML, Stagni AM, Giorgi PP (1994) Accelerated female differentiation of the gonad by inhibition of steroidogenesis in amphibia. Comp Biochem Physiol A 107: 171–179.

[48] Amemiya CT, Alföldi J, Lee AP, Fan S, Brinkmann H, et al.. (2013) The African coelacanth genome provides insights into the tetrapod evolution. Nature (in press). 10.1038/nature12027 PMC3633110 23598338

[49] Pallavicini A, Canapa A, Barucca M, Alföldi J, Biscotti MA, et al. (2013) Analysis of the transcriptome of the Indonesian coelacanth *Latimeria menadoensis*. (submitted). 10.1186/1471-2164-14-538 PMC3750513 23927401

[50] Canapa A, Olmo E, Forconi M, Pallavicini A, Makapedua MD, et al (2012) Composition and phylogenetic analysis of vitellogenin coding sequences in the Indonesian coelacanth *Latimeria menadoensis* . J Exp Zool B Mol Dev Evol 318: 404–416. 22711571 10.1002/jez.b.22455

[51] Makapedua DM, Barucca M, Forconi M, Antonucci N, Bizzaro D, et al (2011) Genome size, GC percentage and 5 mC level in the Indonesian coelacanth *Latimeria menadoensis* . Mar Genomics 4: 167–172. 21867968 10.1016/j.margen.2011.04.001

[52] Grabherr MG, Haas BJ, Yassour M, Levin JZ, Thompson DA, et al (2011) Full-length transcriptome assembly from RNA-Seq data without a reference genome. Nat Biotechnol 29: 644–652. 21572440 10.1038/nbt.1883 PMC3571712

[53] Zhang Z, Li J, Zhao XQ, Wang J, Wong GK, et al (2006) KaKs Calculator: Calculating Ka and Ks through model selection and model averaging. Geno Prot Bioinfo 4: 259–263. 10.1016/S1672-0229(07)60007-2 PMC5054075 17531802

[54] Nei M, Gojobori T (1986) Simple methods for estimating the numbers of synonymous and nonsynonymous nucleotide substitutions. Mol Biol Evol 3: 418–426. 3444411 10.1093/oxfordjournals.molbev.a040410

[55] Tamura K, Peterson D, Peterson N, Stecher G, Nei M, et al (2011) MEGA5: molecular evolutionary genetics analysis using maximum likelihood, evolutionary distance, and maximum parsimony methods. Mol Biol Evol 28: 2731–2739. 21546353 10.1093/molbev/msr121 PMC3203626

[56] Zhang J, Rosenberg HF, Nei M (1998) Positive Darwinian selection after gene duplication in primate ribonuclease genes. Proc Natl Acad Sci USA 95: 3708–3713. 9520431 10.1073/pnas.95.7.3708 PMC19901

[57] Larkin MA, Blackshields G, Brown NP, Chenna R, McGettigan PA, et al (2007) ClustalW and ClustalX version 2 (2007). Bioinformatics 23: 2947–2948. 17846036 10.1093/bioinformatics/btm404

[58] Eisenberg E, Levanon EY (2003) Human housekeeping genes are compact. Trends Genet 19: 362–365. 12850439 10.1016/S0168-9525(03)00140-9

[59] Huelsenbeck JP, Ronquist F, Nielsen R, Bollback JP (2001) Bayesian inference of phylogeny and its impact on evolutionary biology. Science 294: 2310–2314. 11743192 10.1126/science.1065889

[60] Dayhoff M, Schwartz R, Orcutt B (1978) A model of evolutionary change in protein. Atlas Protein Seq Struct 5: 345–352.

[61] Jones DT, Taylor WR, Thornton JM (1992) The rapid generation of mutation data matrices from protein sequences. CABIOS 8: 275–282. 1633570 10.1093/bioinformatics/8.3.275

[62] Swofford DL (2002) PAUP*. Phylogenetic analysis using parsimony (* and other methods) version 4. Sunderland, MA: Sinauer Associates.

[63] Itoh N, Konishi M (2007) The zebrafish FGF family. Zebrafish 4: 179–186. 18041922 10.1089/zeb.2007.0509

[64] Sun YL, Zeng S, Ye K, Yang C, Li MH, et al (2012) Involvement of FGF9/16/20 subfamily in female germ cell development of the Nile tilapia, *Oreochromis niloticus* . Fish Physiol Biochem 38: 1427–1439. 22451340 10.1007/s10695-012-9630-4

[65] Herpin A, Schartl M (2011) *Dmrt1* genes at the crossroads: a widespread and central class of sexual development factors in fish. FEBS J 278: 1010–1019. 21281449 10.1111/j.1742-4658.2011.08030.x

[66] Matson CK, Zarkower D (2012) Sex and the singular DM domain: insights into sexual regulation, evolution and plasticity. Nat Rev Genet 13: 163–174. 22310892 10.1038/nrg3161 PMC3595575

[67] Chue J, Smith CA (2011) Sex determination and sexual differentiation in the avian model. FEBS J 278: 1027–1034. 21281451 10.1111/j.1742-4658.2011.08032.x

[68] Raymond CS, Shamu CE, Shen MM, Seifert KJ, Hirsch B, et al (1998) Evidence for evolutionary conservation of sex-determining genes. Nature 391: 691–695. 9490411 10.1038/35618

[69] Rhen T, Schroeder T (2010) Molecular mechanisms of sex determination in reptiles. Sex Dev 4: 16–28. 20145384 10.1159/000282495 PMC2918650

[70] Anand A, Patel M, Lalremruata A, Singh AP, Agrawal R, et al (2008) Multiple alternative splicing of *DMRT1* during gonadogenesis in Indian mugger, a species exhibiting temperature-dependent sex determination. Gene 425: 56–63. 18775479 10.1016/j.gene.2008.08.005

[71] Guo Y, Cheng H, Huang X, Gao S, Yu H, et al (2005) Gene structure, multiple alternative splicing, and expression in gonads of zebrafish *DMRT1* . Biochem Biophys Res Commun 330: 950–957. 15809088 10.1016/j.bbrc.2005.03.066

[72] Zhao Y, Lu H, Yu H, Cheng H, Zhou R (2007) Multiple alternative splicing in gonads of chicken *DMRT1* . Dev Genes Evol 217: 119–126. 17120025 10.1007/s00427-006-0117-0

[73] Shinomiya A, Otake H, Togashi K, Hamaguchi S, Sakaizumi M (2004) Field survey of sex-reversals in the medaka, *Oryzias latipes*: genotypic sexing of wild populations. Zoolog Sci 21: 613–619. 15226583 10.2108/zsj.21.613

[74] Bogart JP, Balon EK, Bruton MN (1994) The chromosomes of the living coelacanth and their remarkable similarity to those of one of the most ancient frogs. J Hered 85: 322–325. 7930502 10.1093/oxfordjournals.jhered.a111470

[75] Kim Y, Kobayashi A, Sekido R, DiNapoli L, Brennan J, et al (2006) Fgf9 and Wnt4 Act as Antagonistic Signals to Regulate Mammalian Sex Determination. PLoS Biol 4: e187. 16700629 10.1371/journal.pbio.0040187 PMC1463023

[76] Nakatani M, Takehara Y, Sugino H, Matsumoto M, Hashimoto O, et al (2008) Transgenic expression of a myostatin inhibitor derived from follistatin increases skeletal muscle mass and ameliorates dystrophic pathology in mdx mice. FASEB J 22: 477–487. 17893249 10.1096/fj.07-8673com

[77] Higasa K, Nikaido M, Saito TL, Yoshimura J, Suzuki Y, et al (2012) Extremely slow rate of evolution in the HOX cluster revealed by comparison between Tanzanian and Indonesian coelacanths. Gene 505: 324–332. 22698790 10.1016/j.gene.2012.05.047

[78] Johnsen H, Andersen Ø (2012) Sex dimorphic expression of five *dmrt* genes identified in the Atlantic cod genome. The fish-specific *dmrt2b* diverged from *dmrt2a* before the fish whole-genome duplication. Gene 505: 221–232. 22749781 10.1016/j.gene.2012.06.021

[79] Herpin A, Schindler D, Kraiss A, Hornung U, Winkler C, et al (2007) Inhibition of primordial germ cell proliferation by the medaka male determining gene DmrtIbY. BMC Dev Biol 7: 99. 17760954 10.1186/1471-213X-7-99 PMC2034567

[80] Hornung U, Herpin A, Schartl M (2007) Expression of the male determining gene dmrtIbY and its autosomal coorthologue dmrt1a in medaka. Sex Dev 1: 197–206. 18391530 10.1159/000102108

[81] Bratus A, Slota E (2009) Comparative cytogenetic and molecular studies of *DM* domain genes in pig and cattle. Cytogenet Genome Res 126: 180–185. 20016168 10.1159/000245918

[82] Smith CA, Hurley TM, McClive PJ, Sinclair AH (2002) Restricted expression of *DMRT3* in chicken and mouse embryos. Gene Expr Patterns 2: 69–72. 12617839 10.1016/s0925-4773(02)00360-x

[83] Winkler C, Hornung U, Kondo M, Neuner C, Duschl J, et al (2004) Developmentally regulated and non-sex-specific expression of autosomal *dmrt* genes in embryos of the Medaka fish (*Oryzias latipes*). Mech Dev 121: 997–1005. 15210205 10.1016/j.mod.2004.03.018

[84] Kapoor BG, Khanna B (2004) Ichthyology Handbook. Berlin: Springer 487.

[85] Nakamura S, Watakabe I, Nishimura T, Picard JY, Toyoda A, et al (2012) Hyperproliferation of mitotically active germ cells due to defective anti-Müllerian hormone signaling mediates sex reversal in medaka. Development 139: 2283–2287. 22627284 10.1242/dev.076307

[86] Klüver N, Pfennig F, Pala I, Storch K, Schlieder M, et al (2007) Differential expression of anti-Müllerian hormone (*amh*) and anti-Müllerian hormone receptor type II (*amhrII*) in the teleost medaka. Dev Dyn 236: 271–281. 17075875 10.1002/dvdy.20997

[87] Pala I, Klüver N, Thorsteinsdóttir S, Schartl M, Coelho MM (2008) Expression pattern of anti-Müllerian hormone (*amh*) in the hybrid fish complex of *Squalius alburnoides* . Gene 410: 249–258. 18242010 10.1016/j.gene.2007.12.018

[88] Halm S, Rocha A, Miura T, Prat F, Zanuy S (2006) Anti-Müllerian hormone (AMH/AMH) in the European sea bass: its gene structure, regulatory elements, and the expression of alternatively-spliced isoforms. Gene 388: 148–158. 17157448 10.1016/j.gene.2006.10.018

[89] Beau C, Rauch M, Joulin V, Jégou B, Guerrier D (2000) GATA-1 is a potential repressor of anti-Müllerian hormone expression during the establishment of puberty in the mouse. Mol Reprod Dev 56: 124–138. 10813843 10.1002/(SICI)1098-2795(200006)56:2<124::AID-MRD2>3.0.CO;2-J

[90] Ooe H, Chen Q, Kon J, Sasaki K, Miyoshi H, et al (2012) Proliferation of rat small hepatocytes requires follistatin expression. J Cell Physiol 227: 2363–2370. 21826650 10.1002/jcp.22971

[91] Rey R, Lukas-Croisier C, Lasala C, Bedecarrás P (2003) AMH/MIS: what we know already about the gene, the protein and its regulation. Mol Cell Endocrinol 211: 21–31. 14656472 10.1016/j.mce.2003.09.007

[92] El-Awady MK, El-Garf W, El-Houssieny L (2004) Steroid 5alpha reductase mRNA type 1 is differentially regulated by androgens and glucocorticoids in the rat liver. Endocr J 51: 37–46. 15004407 10.1507/endocrj.51.37

[93] Barrionuevo F, Georg I, Scherthan H, Lécureuil C, Guillou F, et al (2009) Testis cord differentiation after the sex determination stage is independent of *Sox9* but fails in the combined absence of *Sox9* and *Sox8* . Dev Biol 327: 301–312. 19124014 10.1016/j.ydbio.2008.12.011

[94] Nakamura S, Watakabe I, Nishimura T, Toyoda A, Taniguchi Y, et al (2012) Analysis of Medaka *sox9* Orthologue Reveals a Conserved Role in Germ Cell Maintenance. PLoS ONE 7: e29982. 22253846 10.1371/journal.pone.0029982 PMC3257256

[95] Bertrand S, Camasses A, Somorjai I, Belgacem MR, Chabrol O, et al (2011) Amphioxus FGF signaling predicts the acquisition of vertebrate morphological traits. Proc Natl Acad Sci USA 108: 9160–9165. 21571634 10.1073/pnas.1014235108 PMC3107284

