# Supplementary figures and images for: Characterization of Sex Determination and Sex Differentiation Genes in Latimeria
Source: PLoS One. 2013 Apr 25;8(4):e56006. doi: 10.1371/journal.pone.0056006 (PMC3636272; doi:10.1371/journal.pone.0056006)

# A

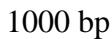

## B Female sex development genes

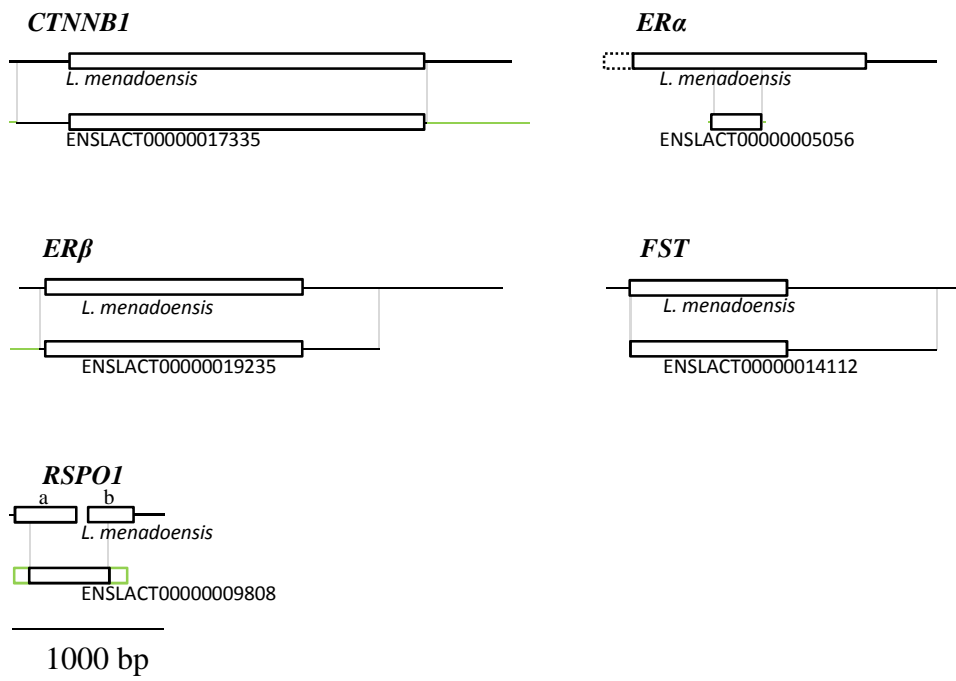

Supplement: Figure S1 — Sequence pair comparison of male sex development genes. Sequence pair comparison of male sex-determining/differentiation transcripts from the L. menadoensis transcriptome and L. chalumnae ENSEMBL predictions. Boxes represent CDSs. Lines represent UTRs. Dashed boxes represent a missing part in the CDS. Green lines/boxes represent an inaccurate gene prediction or a mismatch between L. chalumnae and L. menadoensis sequences. Scale dimension are preserved. B) Sequence pair comparison of female sex development genes. Sequence pair comparison of female sex-determining/differentiation transcripts from the L. menadoensis transcriptome and L. chalumnae ENSEMBL predictions. Boxes represent CDSs. Lines represent UTRs. Dashed boxes represent a missing part in the CDS. Green lines/boxes represent an inaccurate gene prediction or a mismatch between L. chalumnae and L. menadoensis sequences. Scale dimension are preserved. (PDF) [file pone.0056006.s001.pdf]

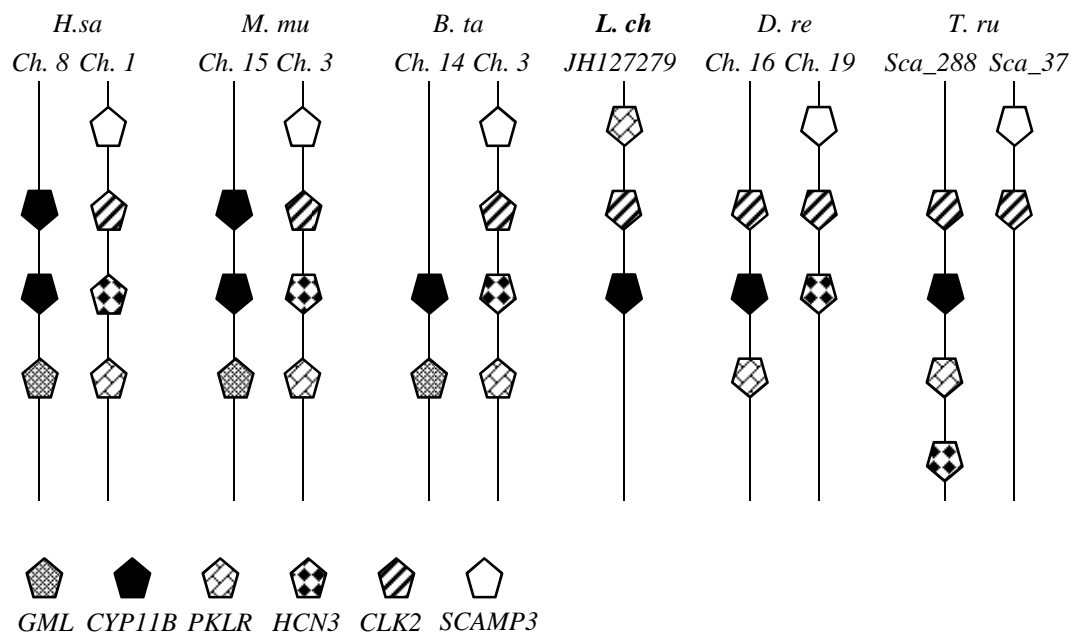

Supplement: Figure S2 — Micro-syntenic conservation of CYP11B . Micro-syntenic conservation of genomic regions containing CYP11B genes. Black pentagons represent CYP11B genes. The pentagon tip points to the relative gene orientation. ENSEMBL data: H. sa (Homo sapiens), M. mu (Mus musculus), B. ta (Bos taurus), L. ch (Latimeria chalumnae), D. re (Danio rerio), T. ru (Takifugu rubripes). (PDF) [file pone.0056006.s002.pdf]
