# Supplementary material for: Characterization of Sex Determination and Sex Differentiation Genes in Latimeria
Source: PLoS One. 2013 Apr 25;8(4):e56006. doi: 10.1371/journal.pone.0056006 (PMC3636272; doi:10.1371/journal.pone.0056006)
Supplement: Table S1 — Gene Ontology analysis of the “sex determination” term. (PDF) [file pone.0056006.s003.pdf]

Table S1 Gene ontologies, comparison with selected vertebrates

**GO0007530 (Sex determination)**	**Total annotations**	**Matching annotations**	***L. menadoensis* orthologs**
Danio rerio	2	2	2
Xenopus laevis	3	3	2
Gallus gallus	27	27	13
Canis familiaris	19	4	4
Sus scrofa	17	19	15
Bos taurus	12	12	11
Mus musculus	21	3	3
Rattus norvegicus	26	25	18
Homo sapiens	33	31	20
