# Supplementary material for: Characterization of Sex Determination and Sex Differentiation Genes in Latimeria
Source: PLoS One. 2013 Apr 25;8(4):e56006. doi: 10.1371/journal.pone.0056006 (PMC3636272; doi:10.1371/journal.pone.0056006)
Supplement: Table S2 — Gene Ontology analysis of the “sex differentiation” term. (PDF) [file pone.0056006.s004.pdf]

Table S2 Gene ontologies, comparison with selected vertebrates

**GO0007548 (Sex differentiation)**	**Total annotations**	**Matching annotations**	***L. menadoensis* orthologs**
Danio rerio	43	43	24
Xenopus laevis	4	4	3
Gallus gallus	187	187	124
Canis familiaris	172	33	28
Sus scrofa	144	143	117
Bos taurus	151	147	118
Mus musculus	203	32	30
Rattus norvegicus	299	291	240
Homo sapiens	373	363	221
